# *Sphingomonas lacusdianchii* sp. nov., an attached bacterium inhibited by metabolites from its symbiotic cyanobacterium

**DOI:** 10.1007/s00253-024-13081-x

**Published:** 2024-04-25

**Authors:** Xin Wang, Yao Xiao, Yang Deng, Xue Sang, Qing-Lin Deng, Le Wang, Yi-Wen Yang, Bing-Huo Zhang, Yu-Qin Zhang

**Affiliations:** 1 College of Pharmacy and Life Science, Jiujiang University, Jiujiang, 332000 China; 2 Institute of Medicinal Biotechnology, Chinese Academy of Medical Sciences & Peking Union Medical College, Beijing, 100050 China

**Keywords:** *Sphingomonas lacusdianchii* sp. nov*.*, *Microcystis*, Attached bacteria

## Abstract

**Abstract:**

An alpha-proteobacterial strain JXJ CY 53^ T^ was isolated from the cyanosphere of *Microcystis* sp. FACHB-905 (MF-905) collected from Lake Dianchi, China. JXJ CY 53^ T^ was observed to be an aerobic, Gram-stain-negative, oval shaped, and mucus-secreting bacterium. It had C_18:1_ω7c and C_16:0_ as the major cellular fatty acids, Q-10 as the predominant ubiquinone, and sphingoglycolipid, diphosphatidylglycerol, phosphatidylcholine, and phosphatidylmethylethanolamine as the polar lipids. The G + C content of DNA was 65.85%. The bacterium had 16S rRNA gene sequence identities of 98.9% and 98.7% with *Sphingomonas panni* DSM 15761^ T^ and *Sphingomonas hankookensis* KCTC 22579^ T^, respectively, while less than 97.4% identities with other members of the genus. Further taxonomic analysis indicated that JXJ CY 53^ T^ represented a new member of *Sphingomonas*, and the species epithet was proposed as *Sphingomonas lacusdianchii* sp. nov. (type strain JXJ CY 53^ T^ = KCTC 72813^ T^ = CGMCC 1.17657^ T^). JXJ CY 53^ T^ promoted the growth of MF-905 by providing bio-available phosphorus and nitrogen, plant hormones, vitamins, and carotenoids. It could modulate the relative abundances of nonculturable bacteria associated with MF-905 and influence the interactions of MF-905 and other bacteria isolated from the cyanobacterium, in addition to microcystin production characteristics. Meanwhile, MF-905 could provide JXJ CY 53^ T^ dissolved organic carbon for growth, and control the growth of JXJ CY 53^ T^ by secreting specific chemicals other than microcystins. Overall, these results suggest that the interactions between *Microcystis* and its attached bacteria are complex and dynamic, and may influence the growth characteristics of the cyanobacterium. This study provided new ideas to understand the interactions between *Microcystis* and its attached bacteria.

**Key points:**

• *A novel bacterium (JXJCY 53* ^*T*^*) was isolated from the cyanosphere of Microcystis sp. FACHB-905 (MF-905)*

• *JXJCY 53* ^*T*^ *modulated the growth and microcystin production of MF-905*

• *MF-905 could control the attached bacteria by specific chemicals other than microcystins (MCs)*

**Supplementary Information:**

The online version contains supplementary material available at 10.1007/s00253-024-13081-x.

## Introduction

Harmful *Microcystis* blooms (HMBs) are one of the most harmful cyanobacterial blooms in freshwater lakes. One of the most serious hazards caused by HMBs is the synthesis and secretion of large amounts of microcystins (MCs), which are mainly produced by *Microcystis aeruginosa* (Dawson 1998), the most common bloom-forming cyanobacterium (Park et al. 2009). Over 270 different MCs have been found up to now (Lin et al. 2021), and the concentrations of microcystin-LR (MC-LR) were the highest in both natural cyanobacterial blooms (Vasconcelos et al. 1996) and *M. aeruginosa* cultures in laboratory (Liu et al. 2012).

Lake Dianchi is the largest lake in the Yunnan-Guizhou Plateau, China. Since the 1980s, cyanobacterial blooms, mainly caused by *M. aeruginosa*, have gradually become a common phenomenon in the lake because of the increasing dumping of various wastes (Liu 1999). Furthermore, this phenomenon worsened from 1990 to 2010, which resulted in more frequent occurrence rates of blooms in this lake. The plankton *Microcystis* can provide special ecological niches for many chemotrophic bacteria (Dziallas and Grossart 2011; Parveen et al. 2013) owing to the extracellular mucous zone mainly composed of a polysaccharide matrix. During the long co-evolution process, various interactions were formed between *Microcystis* and its attached bacteria. Among them, nutrient exchange is one of the most common interactions between photosynthetic cyanobacteria and heterotrophic bacteria (Kouzuma and Watanabe 2015). Soluble organic carbons secreted by cyanobacteria can be used by heterotrophic bacteria for survival, and soluble phosphorus, bio-available nitrogen, vitamins (Yang and Xiao 2011), and indole-3-acetic acid (Hoke et al. 2021) can be supplied to the cyanobacteria by heterotrophic bacteria in return.

During studies of attached bacteria of *Microcystis* sp. FACHB-905 (MF-905), a new alpha-proteobacterial strain JXJ CY 53^ T^, pertaining to the *Sphingomonas*, was isolated from the cyanosphere of MF-905. The genus *Sphingomonas* was originally described by Yabuuchi et al. (1990) and, subsequently, revised by some researchers (Takeuchi et al. 1993, 2001; Pal et al. 2005, 2006), and finally subdivided into four distinct genera: *Sphingomonas* sensu stricto, *Novosphingobium*, *Sphingopyxis*, and *Sphingobium* (Takeuchi et al. 2001). Species of the genus *Sphingomonas* were found in various environments (Madhaiyan et al. 2020), which can degrade hexachlorocyclohexane (Pal et al. 2005), polycyclic aromatic hydrocarbons, and synthetic pesticides (Zhang et al. 2021), and have many other biotechnological applications (Denner et al. 2001; Zhang et al. 2021). Many *Sphingomonas* strains were isolated from cyanobacterial blooms (Berg et al. 2009; Secker et al. 2016; Shao et al. 2014), and different *Sphingomonas* strains probably pose different effects on cyanobacterial growth, including promotion, inhibition, or other effects (Berg et al. 2009). *Sphingomonas* strains can degrade cyanobacterial toxins including microcystins (Berg et al. 2009; Secker et al. 2016) and could be used in assessing and controlling the harmful effects of cyanobacteria (Berg et al. 2009). The genus *Sphingomonas* contained 158 species with validly published names till June 2023 (https://www.bacterio.net/). In this study, the taxonomic status of JXJ CY 53^ T^ was determined using a polyphasic approach and identified as a new member of *Sphingomonas*, and the species epithet was proposed as *Sphingomonas lacusdianchii* sp. nov. The interactions of the bacterium and MF-905 were also investigated by a co-culture method in vitro.

## Materials and methods

### Isolation of bacteria and *Microcystis*

About 0.1 mL MF-905 culture, obtained from Freshwater Algae Culture Collection at the Institute of Hydrobiology, FACHB (https://algae.ihb.ac.cn/), was spread onto sterile Trypticase Soy Agar (TSA) medium plates for isolation of the heterotrophic bacteria. The isolation plates were cultured at 28 °C for 2–8 days, and the colonies of different morphologies and colors were picked and re-streaked repeatedly onto TSA medium to obtain pure cultures. The pure cultures of heterotrophic bacteria were preserved at 4.0 °C using TSA slants and − 80.0 °C using glycerol suspensions (30–50%, v/v), respectively. MF-905 cells were purified by streaking onto the BG11 (Blue-Green Medium) (Allen 1968) agar plates, and the plates were cultured under about 1667 lx illumination on a 12-h light:12 h dark cycle at about 25 °C for 30–60 days. The purified distinct colonies of cyanobacterium were transferred to BG11 liquid medium and cultured under the conditions described above until the liquid medium displayed a green hue. Subsequently, the cultures were assessed for bacterial contamination through spreading onto the TSA plates, which were then cultured at 28 °C for 7 days. The lack of bacterial colonies on these plates confirmed the successful purification of the *Microcystis* sp. FACHB-905 strain, hereafter referred to as purified MF-905.

### Phenotypic features

Cellular morphology was surveyed by using microscopy after the bacterium was cultured on TSA medium at 28.0 °C for 4 days. Gram-staining was done as described by Dong and Cai (2001). Catalase activity was determined as described by Zhang et al. (2017). Ranges of growth temperature, pH, and NaCl contents were determined by using TSA medium as the basic medium. The tests for hydrolysis of Tween 20, 40, 80, and starch, and H_2_S production were done as described by Dong and Cai (2001). Other features of physio-biochemistry were appraised using API 20NE and API 50CH (bioMérieux, Marcy l’Etoile, France).

### Chemotaxonomic characteristics

After being cultured on TSA medium at 28.0 °C for 3 days, cell mass was collected for chemical analysis. Phospholipids were extracted and detected as described by Minnikin et al. (1997). Fatty acids were detected by using the microbial identification system (Sherlock Version 6.1; TSBA6 MIDI 2000; MIDI, Inc., Newark, DE, USA). Respiratory quinones were extracted with a mixture of chloroform and methanol (Collins et al. 1977) and detected using high performance liquid chromatography (HPLC; Tamaoka et al. 1983).

### Phylogenetic and whole genome sequencing analysis

The obtained 16S rRNA gene sequence was submitted to EzBioCloud Databases (Yoon et al. 2017) to be aligned with the available sequences to determine the approximate phylogenetic affiliation of the isolate. Accordingly, the closest relative species with valid names were downloaded. A neighbor-joining tree (Saitou and Nei 1987), a maximum-parsimony tree (Fitch 1971), and a maximum-likelihood tree (Felsenstein 1981) were generated by MEGA version 5.0 (Tamura et al. 2011). The tree topologies were assessed by bootstrap analysis (Felsenstein 1985) of 1000 replicates.

The whole genomes sequencing of JXJ CY 53^ T^ and its two closest reference type strains, *Sphingomonas panni* DSM 15761^ T^ (Busse et al. 2005) and *Sphingomonas hankookensis* KCTC 22579^ T^ (Yoon et al. 2009), was done on the Illumina HiSeq 4000 platform (Illumina Inc., San Diego, CA, USA). The qualities of the raw reads were evaluated and pruned using software of FastQC and Trimmomatic (Bolger et al. 2014), respectively. The second-generation sequence data were assembled using SPAdes software (Bankevich et al. 2012). The resultant assembled contigs were filled for the gaps using GapFiller software (Boetzer and Pirovano 2012) and corrected with PrinSeS-G software (Massouras et al. 2010). The genetic factors were predicted by using Prokka version 1.10 (Seemann 2014). Repeat sequences were confirmed by using RepeatModeler and RepeatMasker (https://www.repeatmasker.org/). Clustered Regularly Interspaced Palindromic Repeats (CRISPR) prediction and analysis were done by using the CRT Tool (Bland et al. 2007). Genomic annotations were done by using the NCBI Blast + with the default arguments. The digital DNA-DNA hybridization (dDDH) level was determined using the Genome-to-Genome Distance Calculator (Meier-Kolthoff et al. 2013). The average nucleotide identity (ANI) was calculated using the JSpeciesWS website (http://jspecies.ribohost.com/jspeciesws/#analyse). The G + C content of DNA was computed based on the genome sequence. Gene prediction and annotation were carried out as described by Chen et al. (2020).

### Capacity to dissolve insoluble phosphorus and fix nitrogen

The capacity of the bacterium to dissolve phytin and tricalcium phosphate (1 g/L) was appraised as described previously (Zhang et al. 2016b). Azotification of the bacterium was confirmed using nitrogen-free medium (Xiao et al. 2022a).

### Co-culture of MF-905 and its attached bacteria

Eight other strains of bacteria (Table 1) isolated from the cyanosphere of MF-905 were used in the co-culture experiments in addition to JXJ CY 53^ T^. Purified MF-905 were co-cultured with attached bacteria as described previously (Xiao et al. 2022b), and the final cellular densities of both MF-905 and bacteria were 1.0 × 10^6^ CFU/mL (colony-forming units/mL). Bibasic co-cultures (BC) consisted of MF-905 and one of the nine bacterial strains, and tribasic co-cultures (TC) consisted of MF-905 and JXJ CY 53^ T^, in addition to one of the other eight bacterial strains (Table 1). Purified MF-905 and bacteria with the same cellular densities were cultured independently in BG11 medium as the controls. Both controls and co-cultures were done in triplicates. The bacterial cellular densities were detected using plate counts on days 5 and 10, respectively. After 5 and 10 days of incubations, both the MF-905 control and co-cultures were centrifuged at 4860 g for 10 min at 20 °C, and the resultant sediments and liquid supernatants were collected, respectively. The sediments were frozen at − 20 °C for 24 h in advance and then mixed with 90% ethanol (85 °C) and extracted in the dark at 20 °C for 4 h before the concentrations of chlorophyll *a* (chl*-a*) and intracellular microcystin LR (I-MC-LR) were detected using spectrophotometry (Zhang et al. 2016a) and HPLC (Zhang et al. 2015), respectively. The liquid supernatants were used to detect extracellular microcystin LR (E-MC-LR) by HPLC (Zhang et al. 2015).

**Table 1:** The most similar type strains with other eight attached bacteria of MF-905

Strains	16S rRNA gene GenBank accession no	Most similar strain	16S rRNA gene GenBank accession no	Similarity (%)
JXJ CY 05	MZ708736	*Brevibacterium epidermidis* NBRC 14811^ T^	BCSJ01000023	99.8
JXJ CY 11	OQ181347	*Pseudomonas oleovorans* ATCC 8062^ T^	DQ842018	99.5
JXJ CY 16	OQ162229	*Agrococcus terreus* DNG5^T^	FJ423764	99.7
JXJ CY 18	MZ708737	*Methylorubrum thiocyanatum* DSM 11490^ T^	AB175646	99.1
JXJ CY 28	MZ541062	*Sphingomonas abaci* C42^T^	AJ575817	99.6
JXJ CY 31	MZ708738	*Deinococcus wulumuqiensis* R12^T^	APCS01000185	99.9
JXJ CY 37	OQ162230	*Mycolicibacterium monacense* DSM 44395^ T^	MVIA01000076	99.8
JXJ CY 57	MZ708739	*Pseudomonas toyotomiensis* DSM 26169^ T^	AB453701	99.9

### Co-culture of MF-905 and JXJ CY 53^ T^ with limited bio-available N and P

MF-905 was co-cultured with JXJ CY 53^ T^ in modified BG11 media. The initial cell densities of MF-905 and bacterium were 5 × 10^5^ CFU/mL and 1 × 10^6^ CFU/mL, respectively. In the modified BG11 media, tricalcium phosphate was used as the only phosphorus source, or no nitrogen source was added (nitrogen-free). MF-905 and the bacterium were cultured independently in the modified BG11 medium as the controls. Cellular densities of bacteria, concentrations of chl-*a*, and MC-LR were detected on days 7 and 14 in nitrogen-free medium, and on days 9 and 18 in tricalcium phosphate medium, respectively.

### Influences of the metabolites from MF-905 on attached bacterial growth

Purified MF-905 culture was distilled at 50 °C under reduced pressure to remove water. The resultant condensate was soaked with mixture solvents of water, methanol, ethanol, and ethyl acetate to extract metabolites of MF-905, which were separated by a C_18_ column using mixture solvents of water–methanol (10–0 → ··· → 0–10, *v*/*v*). The resultant eluates were combined into four fractions (based on the detection results of HPLC), designated as fractions I, II, III, and IV, of which the amounts were 12.5, 1.75, 0.2, and 0.9 g, respectively. Fractions I, II, and III were water-soluble parts. Fraction IV was a fat-soluble one. Only fraction III was detected to contain MC-LR, of which the amount was about 6.2 mg. The inhibitory activities of these fractions on attached bacteria were tested using a paper disk method as described previously (Zhang et al. 2016a). The dosages of fractions I, II, and IV were 4 mg/disk. The disk with fraction III contained 4 μg MC-LR. The total extract from MF-905 was dissolved using deionized water, and the solution was also tested for its antibacterial activities at 4 mg/disk.

### Effects of JXJ CY 53.^T^ on nonculturable attached bacteria of MF-905

Purified MF-905 was co-cultured with JXJ CY 53^ T^ in BG11 medium as described above and sampled on days 5, 10, 15, and 35. The initial cell densities of both microbes were 1.0 × 10^6^ CFU/mL. An MF-905 culture without JXJ CY 53^ T^ served as the control. The V3–V4 region of 16S rRNA genes was used to assess the abundances of different microbes according to method described by Amin et al. (2022).

### Deposit numbers of strains used in this study

Strain JXJ CY 53^ T^ was deposited in the Korean Collection for Type Cultures (KCTC) with accession number of KCTC 72813, and in the China General Microbiological Culture Collection Center (CGMCC) with accession number of CGMCC 1.17657. Strains JXJ CY 05, JXJ CY 11, JXJ CY 16, JXJ CY 18, JXJ CY 28, JXJ CY 31, JXJ CY 37, and JXJ CY 57 were deposited in the China Pharmaceutical Culture Collection (CPCC) with accession numbers of CPCC 206381, CPCC 101681, CPCC 206382, CPCC 101682, CPCC 101683, CPCC 101684, CPCC 206383, and CPCC 101685, respectively.

## Results

### Phenotypic features

**Fig. 1:**
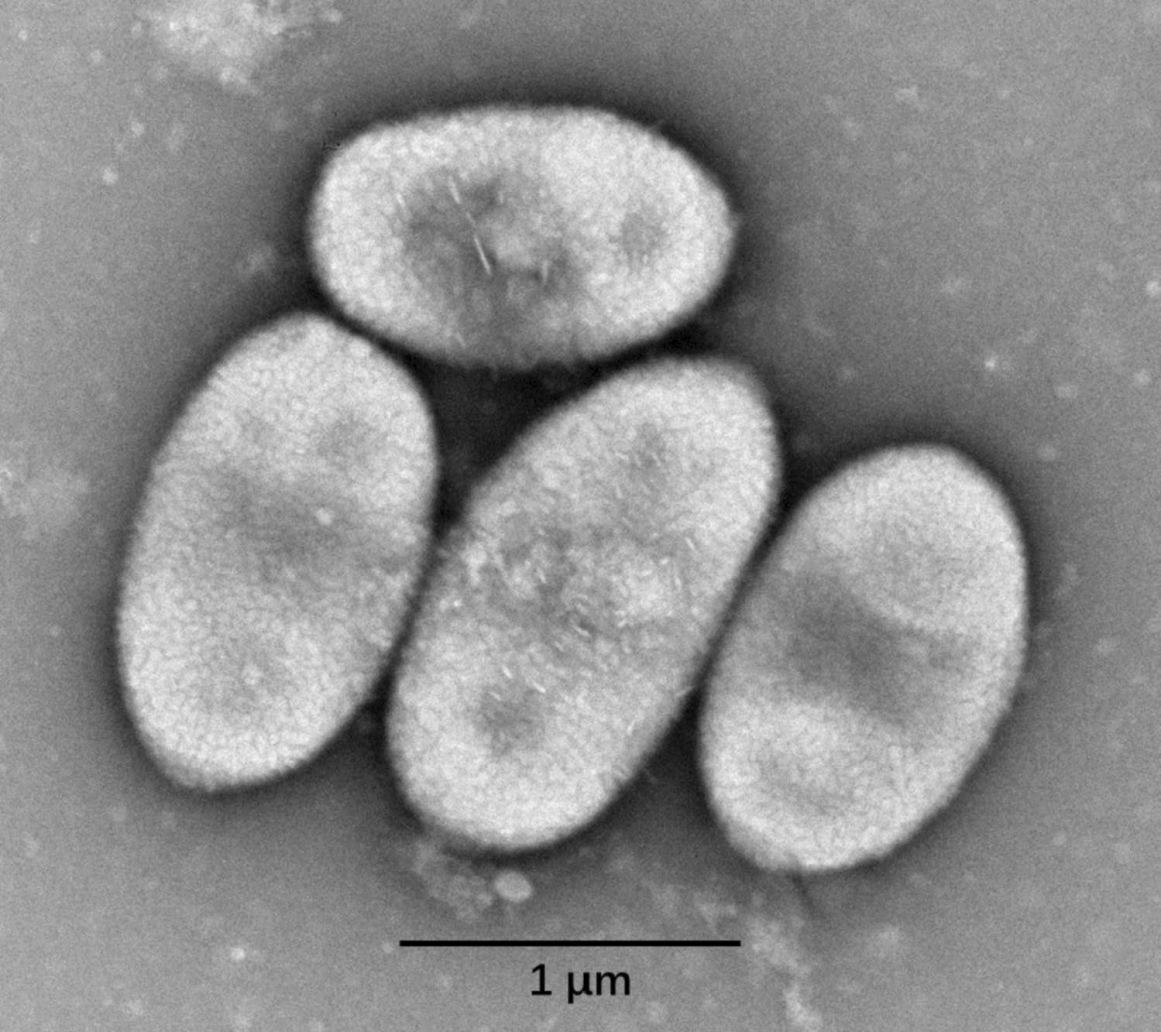
The transmission electron micrograph of strain JXJ CY 53^ T^

Colonies of JXJ CY 53^ T^ were yellow, smooth, circular, and wet in appearance after 3 days of inoculation on TSA plates. Cells were Gram-stain-negative, aerobic, and oval-shaped (0.7–1.0 × 0.9–2.0 μm) (Fig. 1). The growth ranges of pH values, temperatures, and NaCl contents were pH 4.0–11.0, 4.0–40.0 °C, and 0–3.0% (*w*/*v*) NaCl, with optimal ranges of pH 7.0–8.0, 28 °C, and 0% (*w*/*v*) NaCl, respectively. Bubbles arising from the bacterial cell mass showed that its catalase-reaction was positive. After staining with Lugol iodine solution, transparent zones forming around colonies of JXJ CY 53^ T^ grown on starch medium showed that it was positive for starch hydrolysis. Halos formed around colonies of JXJ CY 53^ T^ grown on the media containing Tween 20, 40, and 80, indicating that it could hydrolyze these Tweens. Detailed features were listed in Table 2 with the species description.

**Table 2:** Differential characteristics of strain JXJ CY 53^ T^ and the two reference strains

Characteristic	JXJ CY 53^ T^	*S. panni* DSM 15761^ T^	*S. hankookensis* KCTC 22579^ T^
Isolation source	*Microcystis*	Medical laboratory	Wastewater
Morphology	Oval	Rod	Rod
Colony color	Yellow	Yellow	Yellow
Cell size (µm)	0.7–1.0 × 0.9–2.0	0.6–0.8 × 1.0–2.0	0.4–0.7 × 0.9–2.2
Growth at (°C)	4–40	4–38	10–38
pH range for growth	4.0–11.0	4.0–11.0	4.0–11.0
Tolerance of NaCl (%, w/v)	0–3	0–3	0–2
Catalase	+	+	+
Nitrate reduction	-	-	-
Hydrolysis of:			
Tween 20, 40, 80	+ , + , +	+ , + , +	-, -, -
Starch	Weak	-	-
Oxidase	-	-	+
API 20 NE results			
Glucose	+	-	+
*p*-Nitrophenyl-β-D-galactopyranoside	+	Weak	+
_L_-Arabinose	+	-	+
_D_-Mannose	Weak	-	Weak
Maltose	+	-	+
Gluconate	-	-	+
Adipic acid	-	-	+
_D_-Malate	+	-	+
Citric acid	Weak	-	+
API 50CHB results			
_L_-Arabinose	-	-	Weak
_D_-Xylose	-	-	+
_D_-Galactose	-	-	Weak
_D_-Glucose	-	-	+
Amygdalin	-	-	+
Arbutin	-	-	+
Esculin	+	+	+
Saligenin	-	-	Weak
_D_-Cellobiose	-	-	+
_D_-Maltose	-	-	+
_D_-Lactose	-	-	+
_D_-Saccharose	-	-	+
_D_-Trehalose	-	-	+
_D_-Raffinose	-	-	+
_D_-Fucose	-	-	+

### Chemotaxonomic characteristics

The primary cellular fatty acids were C_18:1_ω7c (48.74%) and C_16:0_ (14.56%), similar to *S. panni* DSM 15761^ T^ and *S. hankookensis* KCTC 22579^ T^ (Supplemental Table S1). Q-10 was its prime ubiquinone. The strain in addition contained diphosphatidylglycerol (DPG), phosphatidylmethylethanolamine (PME), sphingoglycolipid (SGL), phosphatidylcholine (PC), an unidentified lipid (L1), and an unidentified phospholipid (PL) (Supplemental Fig. S1).

### Molecular phylogenetic analysis

**Fig. 2:**
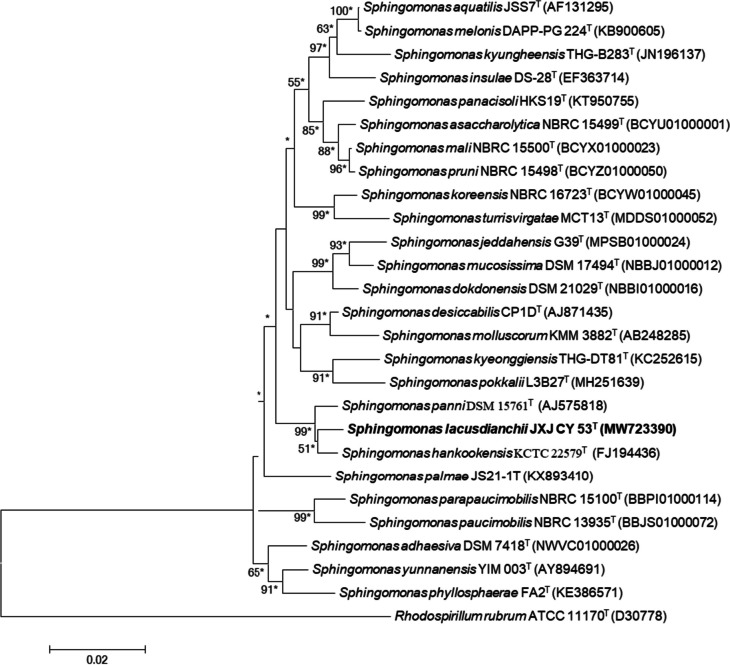
Neighbor-joining phylogenetic tree based on 16S rRNA gene sequences of strain JXJ CY 53^ T^ and its closest relative species of the genus *Sphingomonas*. Bootstrap values (≥ 50%) based on 1000 replications are shown at the branching points. Asterisks indicate that the corresponding nodes were conserved in the trees generated with the maximum-likelihood and maximum-parsimony tree-making algorithms. Bar, 0.02 changes per nucleotide position

Strain JXJ CY 53^ T^ shared high 16S rRNA gene sequence identities with *S. panni* DSM 15761^ T^ (98.9%), *S. hankookensis* KCTC 22579^ T^ (98.7%), and *Sphingomonas desiccabilis* CP1DT (97.3%), while it formed a stable clade with *S. hankookensis* KCTC 22579^ T^ on three different trees (Fig. 2 and Supplemental Figs. S2 and S3). Chun et al. (2018) recommended that ANI and dDDH values needed to be calculated to determine a new taxon only when the 16S rRNA gene sequence identity was higher than 98.7%. In the present study, hence, the ANI and dDDH values were calculated only between JXJ CY 53^ T^ and *S. panni* DSM 15761^ T^ or *S. hankookensis* KCTC 22579^ T^, and they were 83.35% and 29.40%, and 83.02% and 28.90%, respectively, much lower than 95–96% and 70%, the generally accepted species cutoff values (Chun et al. 2018). Hence, combined with other data given above, JXJ CY 53^ T^ was undoubtedly assigned as a novel species of the genus *Sphingomonas*, and the species epithet was proposed as *Sphingomonas lacusdianchii* sp. nov.

### Genomic features

The draft genomes accession numbers of JXJ CY 53^ T^, *S. panni* DSM 15761^ T^, and *S. hankookensis* KCTC 22579^ T^ were JAKRET000000000, JAKREU000000000, and JAKREV000000000, respectively. Features of the established sequence of JXJ CY 53^ T^ were listed in Table 3. Strain JXJ CY 53^ T^ had 1.34% of repeat region, while *S. panni* DSM 15761^ T^ and *S. hankookensis* KCTC 22579^ T^ had no repeat region; and JXJ CY 53^ T^ had 63 tRNA and 5 rRNA gene sequences, while *S. panni* DSM 15761^ T^ and *S. hankookensis* KCTC 22579^ T^ had 59 tRNA and 3 rRNA gene sequences, 60 tRNA and 3 rRNA gene sequences, respectively.

**Table 3:** Genomic features and comparison between strain JXJ CY 53^ T^ and the reference strains

Attribute	JXJ CY 53^ T^	*S. panni* DSM 15761^ T^	*S. hankookensis* KCTC 22579^ T^
GenBank accession no	JAKRET000000000	JAKREU000000000	JAKREV000000000
Genomic size (bp)	4,807,458	4,151,858	3,977,879
Number of contigs	89	28	36
N_50_ length (bp)	244,931	338,320	829,701
Protein coding genes	4460	3922	3764
Coding ratio (%)	88.73	89.44	89.55
Average bp per protein	956.38	946.80	946.40
Repeat region count/rate (%)	1.34	0	0
tRNA	63	59	60
rRNA	5	3	3
G + C content (mol%)	65.85	66.47	66.79
dDDH/ANI value to JXJ CY 53^ T^ (%)		29.40/83.35	28.90/83.02

JXJ CY 53^ T^ harbored genes that facilitated the reciprocity and mutual benefit with the cyanobacterial cells (Supplemental Table S2-S4), including genes involved in coexisting with the cyanobacterium (Supplemental Table S2), exchange of nutrients, synthesis of plant growth hormones, vitamins, and carotenoids (Supplemental Table S3), dissolving insoluble phosphorus and fixing nitrogen (Supplemental Table S4), etc. Meanwhile, metabolism-related genes were retrieved from the genomes of JXJ CY 53^ T^, *S. panni* DSM 15761^ T^, and *S. hankookensis* KCTC 22579^ T^, such as phosphate assimilation-related genes (*phoB*, *phoR*, *pstA*, *pstB*, *pstC*, and *pstS*) and nitrogen fixing genes (*glnA*, *glnB*, *glnD*, *ntrX*, and *ntrY*) (Supplemental Fig. S4).

Phytoene desaturase can catalyze the four-step desaturation of phytoene with the resultant product of lycopene, a kind of carotenoid (Fournié and Truan 2020). Here, the gene for the phytoene desaturase CrtI was detected in all of the genomes of strains JXJ CY 53^ T^, *S. panni* DSM 15761^ T^, and *S. hankookensis* KCTC 22579^ T^. In addition, the potential for four-step desaturations which desaturated phytoene to lycopene was detected in all of these bacteria (Supplemental Fig. S5).

### Capacity to dissolve insoluble phosphorus and fix nitrogen

After 2 days of cultivation in a nitrogen-free medium, living cellular density increased to 2.5 × 10^7^ CFU/mL from 1.0 × 10^6^ CFU/mL. Accordingly, we supposed that JXJ CY 53^ T^ might convert N_2_ into NH_3_ to support its growth. After 2 days of cultivation, soluble phosphorus elements increased by 6.05 ± 0.35 mg/L in tricalcium phosphate medium and 4.59 ± 0.30 mg/L in phytin medium. Resumptively, JXJ CY 53^ T^ might convert insoluble organic and inorganic phosphorus into soluble phosphorus.

### Effects of co-culture on the growths of MF-905 and its attached bacteria

**Fig. 3:**
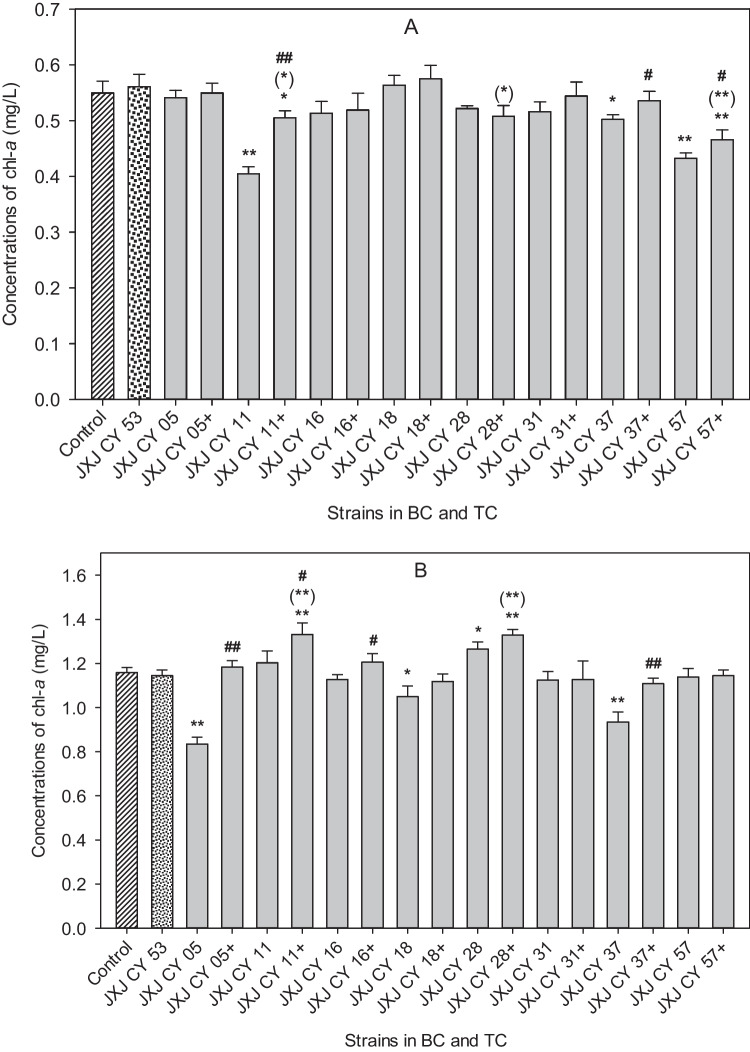
Influences of BC and TC on the growth of MF-905. + , adding JXJ CY 53^ T^. Error bars indicate standard deviations for the three replicates. **A** and **B** were samples of days 5 and 10, respectively. * and ** indicated the significant differences between control and BCs (or TCs) at the levels of *p* < 0.05 and *p* < 0.01, respectively. (*), (**) indicated the significant differences between BC with JXJ CY 53^ T^ and TC at the levels of *p* < 0.05 and *p* < 0.01, respectively. # and ## indicated the significant differences between relevant BC and TC with eight other strains at the levels of *p* < 0.05 and *p* < 0.01, respectively

On days 5 and 10 of cultivation, chl-*a* concentrations in the MF-905 control group increased to 0.550 and 1.158 mg/L from 0.092 mg/L, respectively. In the 5-day cultures, six bacterial strains in BCs exhibited no effects on the biomass of MF-905 (*p* > 0.05) in contrast to JXJ CY 11, 37, and 57, which resulted in the decreases of chl-*a* concentrations by 26.4, 8.6, and 21.3% (*p* < 0.05, *p* < 0.01), respectively (Fig. 3A). Chl-*a* concentrations of TCs were similar to that of the control (*p* > 0.05) except TCs with JXJ CY 53 + 11 and 53 + 57, of which the chl-*a* concentrations decreased by 8.1, and 15.2% (*p* < 0.05, *p* < 0.01) compared with that of the control (Fig. 3A). Chl-*a* concentrations of TCs with JXJ CY 53 + 11, 53 + 37, and 53 + 57 were 0.505, 0.536, and 0.466 mg/L, which were 24.8, 6.7, and 7.7% higher (*p* < 0.05, *p* < 0.01) than those of BCs with JXJ CY 11, 37, and 57 (Fig. 3A), respectively. However, chl-*a* concentrations of TCs with JXJ CY 53 + 11, 53 + 28, and 53 + 57 decreased by 10.0, 9.5, and 16.9% (*p* < 0.05, *p* < 0.01) when compared with that of BC with JXJ CY 53 (Fig. 3A).

On day 10 of co-cultivation, chl-*a* concentrations of BCs with JXJ CY 05, 18, and 37 were 0.834, 1.049, and 0.935 mg/L, which decreased by 28.0, 9.4, and 19.3% (*p* < 0.05, *p* < 0.01), respectively, when compared with that of the control (Fig. 3B). In contrast, the chl-*a* concentration of BC JXJ CY 28 was 1.264 mg/L, which increased by 9.2% (*p* < 0.05) when compared with that of the control (Fig. 3B). On day 10 of co-cultivation, six out of eight TCs did not exhibit an effect on the biomass of MF-905 (*p* > 0.05) except TCs with JXJ CY 53 + 11 and 53 + 28, of which the chl-*a* concentrations were 1.331 and 1.328 mg/L, that increased by 14.9 and 14.7% (*p* < 0.01) when compared with that of the control (Fig. 3B). Chl-*a* concentrations of TCs with JXJ CY 53 + 05, 53 + 11, 53 + 16, and 53 + 37 were 1.183, 1.331, 1.205, and 1.108 mg/L, which increased by 41.8, 10.7, 6.9, and 18.5% (*p* < 0.05, *p* < 0.01), respectively, when compared with those of the relevant BCs (Fig. 3B). Chl-*a* concentrations of TCs with JXJ CY 53 + 11 and 53 + 28 increased by 16.3 and 16.1% (*p* < 0.01), respectively, when compared with that of BC JXJ CY 53.

After 5 days of co-cultivation (Table 4), cellular densities of JXJ CY 16, 28, and 31 in BCs decreased significantly (*p* < 0.01), while that of JXJ CY53, 05, 11, 37, and 57 in BCs increased significantly (*p* < 0.01); cellular densities of JXJ CY 28 and 31 in TCs, and JXJ CY 53^ T^ in TCs with JXJ CY 11, 18, 28, and 57 decreased significantly (*p* < 0.01), while that of JXJ CY 05, 11, 16, 18, 37, and 57 in TCs, and JXJ CY 53^ T^ in TCs with JXJ CY 05, 16, 31, and 37 increased significantly (*p* < 0.05, *p* < 0.01). Then, the densities of these bacteria generally decreased significantly with the culture times in both BCs and TCs (*p* < 0.01). The addition of different bacteria into the BC with JXJ CY 53^ T^ resulted in different effects on the growth of JXJ CY 53^ T^, including inhibiting, promoting (*p* < 0.05, *p* < 0.01), or showing no influences (*p* > 0.05). The addition of JXJ CY 53^ T^ into the BCs with any other of the eight bacteria resulted in similar phenomena.

**Tabel 4:** Cellular densities of nine strains in BC and TC

Strains JXJ CY	Cellular densities (CFU/mL)					
	BC		TC		JXJ CY 53 in TC	
	Day 5	Day 10	Day 5	Day 10	Day 5	Day 10
53	4.53 ± 0.42 × 10^6**^	4.03 ± 0.51 × 10^3**^				
05	9.90 ± 1.41 × 10^6**^	8.53 ± 0.91 × 10^5**^	1.58 ± 0.13 × 10^7**##^	3.02 ± 0.15 × 10^4**##^	1.81 ± 0.21 × 10^6**##^	3.13 ± 0.40 × 10^3**^
11	1.79 ± 0.04 × 10^7**^	3.60 ± 0.66 × 10^6**^	1.63 ± 0.16 × 10^8**##^	1.21 ± 0.12 × 10^6**##^	3.37 ± 0.57 × 10^4**##^	1.57 ± 0.15 × 10^2**##^
16	5.33 ± 0.51 × 10^5**^	4.67 ± 0.58 × 10^2**^	6.20 ± 0.36 × 10^6**##^	3.63 ± 0.51 × 10^3**##^	1.57 ± 0.25 × 10^6*##^	6.13 ± 0.32 × 10^3**##^
18	1.11 ± 0.12 × 10^6^	1.54 ± 0.16 × 10^4**^	2.01 ± 0.32 × 10^7**##^	1.81 ± 0.23 × 10^4**^	7.43 ± 0.70 × 10^5**##^	1.58 ± 0.14 × 10^4**##^
28	5.33 ± 0.76 × 10^4**^	1.77 ± 0.12 × 10^4**^	4.63 ± 0.61 × 10^4**^	5.03 ± 0.57 × 10^4##^	6.47 ± 0.96 × 10^5**##^	3.23 ± 0.23 × 10^3**^
31	1.06 ± 0.12 × 10^5**^	0^**^	0^##^	0	4.23 ± 0.65 × 10^6**^	3.37 ± 0.75 × 10^3**^
37	4.23 ± 0.32 × 10^7**^	1.13 ± 0.14 × 10^6**^	5.33 ± 0.61 × 10^6**##^	2.27 ± 0.22 × 10^5**##^	3.03 ± 0.64 × 10^6**#^	1.17 ± 0.21 × 10^5**##^
57	3.18 ± 0.15 × 10^7**^	1.38 ± 0.06 × 10^7**^	1.61 ± 0.08 × 10^8**##^	7.67 ± 0.64 × 10^6**##^	3.33 ± 0.67 × 10^4**##^	0^**##^

^*^ and ^**^ indicate statistically significant differences of measurements between days 0 and 5 or days 5 and 10 of cultivation in BC (or TC) at the levels of *p* < 0.05 and *p* < 0.01, respectively. ^#^ and ^##^ indicate statistically significant differences between measurements from relevant BC and TC at the levels of *p* < 0.05 and *p* < 0.01, respectively. The initial cellular densities (day 0) of the nine bacteria were all about 1.0 × 10^6^ CFU/mL

### Effects of co-culture on MC-LR concentration

**Fig. 4:**
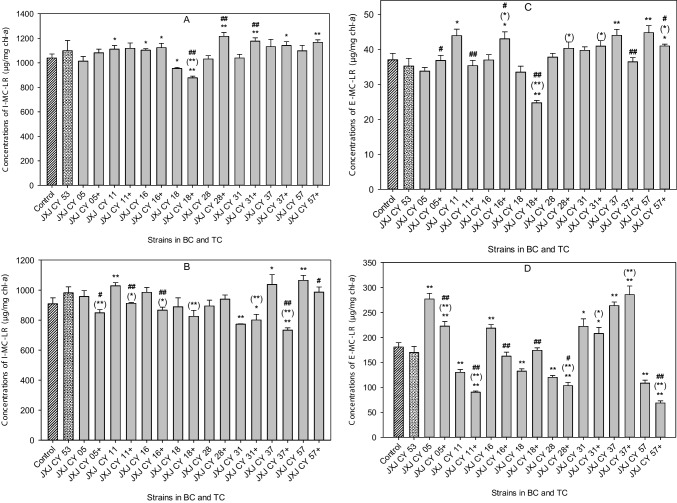
Influences of BC and TC on E-MC-LR and I-MC-LR contents of MF-905. + , adding JXJ CY 53^ T^. Error bars indicate standard deviations for the three replicates. **A** and **C** were samples of day 5; **B** and **D** were samples of day 10. * and ** indicated the significant differences between control and BC (or TC) at the levels of *p* < 0.05 and *p* < 0.01, respectively. (*) and (**) indicated the significant differences between BC with JXJ CY 53^ T^ and TC at the levels of *p* < 0.05 and *p* < 0.01, respectively. # and ## indicated the significant differences between relevant BC and TC at the levels of *p* < 0.05 and *p* < 0.01, respectively

The I-MC-LR concentrations of the control were 1038.9 and 908.1 µg/mg chl-*a* on days 5 and 10 of cultivation, respectively. On day 5 of co-cultivation, I-MC-LR concentrations of BCs with JXJ CY 11, 16, and 18, increased by 6.9 and 6.2%, and decreased by 8.2%, respectively, when compared with that of the control (*p* < 0.05); and I-MC-LR concentrations of TCs with JXJ CY 18 + 53, 28 + 53, 31 + 53, 37 + 53, and 57 + 53 decreased by 15.6% (*p* < 0.01), and increased by 17.1, 13.2, 9.8, and 12.3% (*p* < 0.05, *p* < 0.01), respectively, when compared with that of the control (Fig. 4A). On day 10 of co-cultivation, I-MC-LR concentrations of BCs with JXJ CY 11, 37, 57, and 31 increased by 13.3, 14.2, and 8.6% (*p* < 0.05, *p* < 0.01), and decreased by 14.9% (*p* < 0.01), respectively, when compared with that of the control; and I-MC-LR concentrations of TCs with JXJ CY 31 + 53 and 37 + 53 decreased by 11.8 and 19.3% (*p* < 0.05, *p* < 0.01), respectively, when compared with that of the control (Fig. 4B).

The E-MC-LR concentrations of the control were 37.0 and 180.8 µg/mg chl-*a* on days 5 and 10 of cultivation, respectively. On day 5 of co-cultivation, E-MC-LR concentrations of BCs with JXJ CY 11, 37, and 57 increased by 18.6, 18.9, and 21.0% (*p* < 0.05, *p* < 0.01), respectively, when compared with that of the control; E-MC-LR concentrations of TCs with JXJ CY 53 + 16, 53 + 57, and 53 + 18 increased by 16.2 and 10.6% (*p* < 0.05), and decreased by 33.2% (*p* < 0.01), respectively, when compared with that of the control (Fig. 4C). On day 10 of co-cultivation, E-MC-LR concentrations of BCs with JXJ CY 05, 16, 31, and 37 increased by 53.2, 20.8, 22.9, and 45.9% (*p* < 0.05, *p* < 0.01), respectively, when compared with that of the control, while E-MC-LR concentrations of BCs with JXJ CY 11, 18, 28, and 57 decreased by 28.0, 26.7, 33.5, and 40.0% (*p* < 0.01), respectively, when compared with that of the control (Fig. 4D). On day 10 of co-cultivation, E-MC-LR concentrations of TCs with JXJ CY 53 + 05, 53 + 31, and 53 + 37 increased by 23.1, 15.1, and 58.1% (*p* < 0.05, *p* < 0.01), respectively, when compared with that of the control, while E-MC-LR concentrations of TCs with JXJ CY 53 + 11, 53 + 28, and 53 + 57 decreased by 50.0, 42.9, and 61.9% (*p* < 0.01), respectively, when compared with that of the control (Fig. 4D).

### Effects of limited bio-available N and P on the growth of MF-905 and JXJ CY 53^ T^

In tricalcium phosphate medium, the inoculation of JXJ CY53^T^ significantly influenced the growth and MC-LR synthesis of MF-905 (Table 5). The chl-*a* concentrations of MF-905 co-cultured with JXJ CY53^T^ were 0.826 and 1.074 mg/L on days 9 and 18 and increased by 35.2 and 25.0% (*p* < 0.01), respectively, when compared with those of the controls. The I-MC-LR concentrations of MF-905 co-cultured with JXJ CY53^T^ were 914.2 and 1517.9 μg/mg chl-*a* on days 9 and 18 and decreased by 14.5 and 18.3% (*p* < 0.05, *p* < 0.01), respectively, when compared with those of the controls. The E-MC-LR concentration of MF-905 co-cultured with JXJ CY53^T^ was 19.6 μg/mg chl-*a* on day 18 and decreased by 53.3% (*p* < 0.01) when compared with that of the control.

**Table 5:** Influences of limited available P on the growths of MF-905 and JXJ CY 53^ T^

Groups	Targets	On day 9	On day 18
a	Chl-*a* (mg/L)	0.611 ± 0.008	0.859 ± 0.026
	E-MC-LR (μg/mg chl-*a*)	5.6 ± 2.5	41.9 ± 3.7
	I-MC-LR (μg/mg chl-*a*)	1069.3 ± 44.7	1857.3 ± 85.6
b	Chl-*a* (mg/L)	0.826 ± 0.035^**^	1.074 ± 0.017^**^
	E-MC-LR (μg/mg chl-*a*)	4.1 ± 0.9	19.6 ± 1.0^**^
	I-MC-LR (μg/mg chl-*a*)	914.2 ± 61.2^*^	1517.9 ± 22.8^**^
	Cellular densities of JXJ CY 53^ T^ (CFU/mL)	1.03 ± 0.32 × 10^3##^	8.83 ± 0.40 × 10^4##(**)^
c	Cellular densities of JXJ CY 53^ T^ (CFU/mL)	1.82 ± 0.16 × 10^6^	5.83 ± 0.40 × 10^4(**)^

a, MF-905 cultured with no strain JXJ CY 53^ T^; b, MF-905 cocultured with strain JXJ CY 53^ T^; c, strain JXJ CY 53^ T^ cultured with no MF-905. ^*^, ^**^ indicate the significant differences between a and b at the levels of *p* < 0.05, *p* < 0.01, respectively. ^(**)^ indicated the significant differences of the bacterial cellular densities between day 9 and day 18 at the level of *p* < 0.01. ^##^ indicate the significant differences of the bacterial cellular densities between b and c at the level of *p* < 0.01. The initial cellular density (day 0) of strain JXJ CY 53^ T^ was 1.0 × 10^6^ CFU/mL

In bio-available nitrogen-limited medium, the inoculation of JXJ CY53^T^ also influenced the growth and MC-LR synthesis of MF-095 (Table 6). The chl-*a* concentrations of MF-905 co-cultured with JXJ CY53^T^ were 0.315 mg/L on day 14 and increased by 61.5% (*p* < 0.01) when compared with that of the control. The I-MC-LR concentration of MF-905 co-cultured with JXJ CY53^T^ was 553.0 μg/mg chl-*a* on day 7 and decreased by 14.0% (*p* < 0.01) when compared with that of the control. The E-MC-LR concentration of MF-905 co-cultured with JXJ CY53^T^ was 113.2 μg/mg chl-*a* on day 14 and decreased by 76.2% (*p* < 0.01) when compared with that of the control.

**Table 6:** Influences of limited available N on the growths of MF-905 and JXJ CY 53^ T^

Groups	Targets	On day 7	On day 14
a	Chl-*a* (mg/L)	0.446 ± 0.013	0.195 ± 0.013
	E-MC-LR (μg/mg chl-*a*)	23.3 ± 4.9	474.9 ± 43.4
	I-MC-LR (μg/mg chl-*a*)	642.8 ± 9.3	1002.2 ± 42.1
b	Chl-*a* (mg/L)	0.452 ± 0.008	0.315 ± 0.010^**^
	E-MC-LR (μg/mg chl-*a*)	24.7 ± 17.9	113.2 ± 11.4^**^
	I-MC-LR (μg/mg chl-*a*)	553.0 ± 19.5^**^	936.1 ± 40.1
	Cellular densities of JXJ CY 53^ T^ (CFU/mL)	2.87 ± 0.64 × 10^4##^	2.77 ± 0.45 × 10^6##(**)^
c	Cellular densities of JXJ CY 53^ T^(CFU/mL)	1.61 ± 0.12 × 10^6^	1.22 ± 0.13 × 10^5(**)^

a, MF-905 cultured with no strain JXJ CY 53^T^; b, MF-905 cocultured with strain JXJ CY 53^T^; c, strain JXJ CY 53^T^ cultured with no MF-905. ^**^ indicate the significant differences between a and b at the level of *p* < 0.01, respectively. ^(**)^ indicated the significant differences of the bacterial cellular densities between day 7 and day 14 at the level of *p* < 0.01. ^##^ indicate the significant differences of the bacterial cellular densities between b and c at the level of *p* < 0.01. The initial cellular density (day 0) of strain JXJ CY 53^T^ was 1.0 × 10^6^ CFU/mL

The cellular densities of JXJ CY53^T^ cultured with no MF-905 did not change in the samples collected at the first time for both media, followed by decreasing to about 10^4^–10^5^ CFU/mL (Table 5 and 6). However, the cellular densities of JXJ CY53^T^ co-cultured with MF-905 decreased to 10^3^–10^4^ CFU/mL in the samples collected at the first time, and then increased to 8.83 × 10^4^ CFU/mL in tricalcium phosphate medium, and 2.77 × 10^6^ CFU/mL in bio-available nitrogen-limited medium.

### Inhibitory activity of the metabolites from MF-905 on the attached bacteria

Antibacterial assays showed that the extracts from MF-905 exhibited obvious inhibitory activities on JXJ CY 16, 31, 37, and 53, and no or weak inhibitory activities on other attached bacterial strains (Supplemental Fig. S6). Fraction IV showed no inhibitory activity on all attached bacteria. Fraction II showed the strongest inhibitory activity with inhibition zone diameters of 0.8–1.6 cm on JXJ CY 16, 31, 37, and 53, followed by fraction I. Fraction III, containing MC-LR at 4 μg/disk, showed almost non-inhibitory activities on all of these bacteria.

### Effects of JXJ CY 53.^T^ on nonculturable attached bacteria of MF-905

The data of Illumina MiSeq sequencing showed that relative abundances of MF-905 cultured without JXJ CY 53^ T^ were more than 99.9% on days of 5, 10, and 15, and decreased to 98.35% on day 35. Many other bacteria belonging to different phyla were also detected with relative abundances of 0.0028–0.05% except *Pseudomonadota*, of which the relative abundance could reach 1.59% on day 35. This was due to the relative abundance of *Brevundimonas* affiliated to the *Pseudomonadota* that increased from 0 on day 5 to 1.59% on day 35. The relative abundance of *Lactobacillus* affiliated to the *Bacillota* increased from 0 on day 5 to 0.0265% on day 15, and then it reduced to 0.0118% on day 35. Furthermore, the relative abundance of *Mucilaginibacter* which belongs to the *Bacteroidota* increased from 0 on day 5 to 0.0079% on day 15, and then it reduced to 0.0039% on day 35. However, the relative abundance of *Dubosiella* which belongs to the *Bacillota* reduced from 0.0031% on day 10 to 0 on day 35. Similarly, the relative abundance of *Akkermansia* which belongs to the *Verrucomicrobiota* reduced from 0.0071% on day 10 to 0 on day 35.

JXJ CY 53^ T^ co-cultured with MF-905 in BG11 medium did not die out 35 days later*.* The relative abundances of MF-905 co-cultured with JXJ CY 53^ T^ were 86.90, 52.35, 92.96, and 98.46% on days 5, 10, 15, and 35, respectively; meanwhile, the relative abundances of JXJ CY 53^ T^ co-cultured with MF-905 were 12.25, 17.42, 0.92, and 1.46% on days 5, 10, 15, and 35, respectively. However, the growth trends of nonculturable attached bacteria changed significantly in MF-905 cultures inoculated with JXJ CY 53^ T^. The relative abundance of *Brevundimonas* affiliated to the *Pseudomonadota* increased to 0.005% on day 35, which was much lower than when MF-905 was cultured without JXJ CY 53^ T^. The relative abundance of *Lactobacillus* affiliated to the *Bacillota* increased from 0.0115% on day 5 to 0.0139% on day 10, and then it reduced from 0.0086% on day 15 to 0.0046% on day 35. In addition, the relative abundances of *Mucilaginibacter* which belongs to the *Bacteroidota* were 0.0000, 0.0028, 0.0000, and 0.0054% on days 5, 10, 15, and 35, respectively. However, the relative abundance of *Dubosiella* which belongs to the *Bacillota* was 0.0106, 0.0000, 0.0086, and 0.0093% on days 5, 10, 15, and 35, respectively. Besides, the relative abundance of *Akkermansia* which belongs to the *Verrucomicrobiota* was 0.0048, 0.0111, 0.0034, and 0.0000% on days 5, 10, 15, and 35, respectively. The change of the growth state of these bacteria with the prolonged culture time of MF-905 co-cultured with JXJ CY 53^ T^ was completely different from that of MF-905 cultured without JXJ CY 53^ T^.

In addition, the relative abundances of *Mycobacterium* affiliated to the *Actinomycetota* were 0.80, 30.17, 6.05, and 0.00% on days 5, 10, 15, and 35, respectively, which was totally undetected in MF-905 cultured without JXJ CY 53^ T^. Similarly, the relative abundances of *Pseudomonas* affiliated to the *Pseudomonadota* were 0.017, 0.028, 0.017, and 0.00% on days 5, 10, 15, and 35, respectively, which was also undetected in MF-905 cultured without JXJ CY 53^ T^. Therefore, the growth states of nonculturable attached bacteria of MF-905 were influenced greatly by the inoculation of JXJ CY 53^ T^.

### Description of *Sphingomonas lacusdianchii *sp. nov*.*

*Sphingomonas lacusdianchii* sp. nov. (la.cus.di.a'n.chii L. gen. n. *lacus*, of a lake; N.L. gen. n. *dianchii*, of Dianchi; N.L. gen. n. *lacusdianchii*, of Dianchi lake).

Cells are aerobic, Gram-stain-negative, and oval-shaped (0.7–1.0 × 0.9–2.0 μm), and grow well on TSA medium and secrete mucus. The growth ranges of temperatures, pH values, and NaCl contents are 4.0–40.0 °C, pH 4.0–11.0, and 0–3.0% (*w*/*v*) NaCl, respectively, with optimal values of 28 °C, pH 7.0–8.0, and 0% (*w*/*v*) NaCl. It can hydrolyze starch, Tweens (20, 40, and 80), and produce catalase. But it shows no activity in oxidase and nitrate reduction. C_16:0_ and C_18:1_ω7c are the primary cellular fatty acids. The prime ubiquinone is Q-10. It contains diphosphatidylglycerol (DPG), phosphatidylcholine (PC), phosphatidylmethylethanolamine (PME), sphingoglycolipid (SGL), an unidentified lipid (L1), and an unidentified phospholipid (PL).

The type strain, JXJ CY 53^ T^ (= KCTC 72813^ T^ = CGMCC 1.17657^ T^), was isolated from the cyanosphere of *Microcystis* sp. FACHB-905, collected in Lake Dianchi Yunnan province, China. The GenBank accession numbers for the 16S rRNA gene sequence and draft genome sequence of JXJ CY 53^ T^ are MW723390 and JAKRET000000000, respectively.

## Discussion

Harmful cyanobacterial blooms have become one of the most serious pollution problems in aquatic environment (Zhang et al. 2015; Žegura et al. 2011). However, it is difficult to control the occurrence of cyanobacteria by conventional approaches (Ozaki et al. 2008). The interactions between the cyanobacteria and their attached bacteria have a key impact on the occurrence or duration of the cyanobacterial blooms (Shao et al. 2014). Therefore, the significances of cyanobacterial-attached heterotrophic bacteria must be taken into account when looking for countermeasures to control eutrophic cyanobacterial blooms (Yang and Xiao 2011).

This study used polyphasic systematics to identify the bacterium JXJ CY 53^ T^, isolated from the cyanosphere of MF-905, as a new member of *Sphingomonas*, and the species epithet was proposed as *S. lacusdianchii* sp. nov. Co-cultures of attached bacteria and the cyanobacterium were used to study the interactions between the bacteria and MF-905. The antibacterial assay of the metabolites produced by MF-905 was also done to reveal how the healthy MF-905 controls its attached bacteria. This study provided unique insight regarding the interactions between attached bacteria and *M*. *aeruginosa*, which would probably further provide new clues of controlling cyanobacterial blooms.

### Methodological considerations

The coexistence of bacteria and cyanobacteria has existed ever since the early evolutionary stage (Ramanan et al. 2016). Different cyanobacteria secrete unique exudates, and therefore, only individual bacteria can coexist with such specific cyanobacteria (Yang et al. 2017). The interconnected evolutionary history of cyanobacteria and bacteria allows the formation of various complex interactions between cyanobacteria and heterotrophic bacteria, like trophallaxis, signals transduction, and transgenosis (Kouzuma and Watanabe 2015). Metagenomes, metaproteomes, and metatranscriptomes are the main ways to illustrate the interactions of phytoplankton and heterotrophic bacteria, and these methods can confirm many species existing in the environment and provide the holistic metabolic abilities of the community studied (Kazamia et al. 2016). Few of these studies were done in axenic conditions, which resulted in most of these interaction features (Grossart and Simon 2007) and bacterial ecological functions (Zhang et al. 2019) unknown, especially the exact interactions of these microbes (Zhu et al. 2019). Co-cultures of cyanobacteria and specific bacteria well-characterized under laboratory conditions are offering the foundation to develop ecological principles that represent the microbial community dynamics and lifestyle, and can reveal the specific interactions at the cellular and molecular levels (Kazamia et al. 2016). Therefore, only combined with the defined co-cultures in the laboratory, transcriptomic, metagenomic, and metabolomic approaches can deepen our cognition on these interactions preferably.

### Nutrient exchange between MF-905 and its attached bacteria

The ATP-binding cassette (ABC) transporter complexes are responsible for the transport of many metabolites into and out of the cell. Protein secretion system complexes are secretory proteins that carry out secretion of the cell or mediate the movement of proteins into the extracellular environment. Both ABC transporter and protein secretion systems participate in the signal transductions and material exchanges between the cyanobacteria and heterotrophic bacteria (Zhu et al. 2021). JXJ CY 53^ T^ has four genes related to ABC transporter and 23 genes related to protein secretion systems (Supplemental Table S3), which means that there are potential signal transductions and material exchanges between MF-905 and JXJ CY 53^ T^, such as exchanges of nutrients, auxin-mediated signaling pathway, and blue light signaling pathway. Nutrient exchange is the most common interaction between the cyanobacteria and the heterotrophic bacteria (Kouzuma and Watanabe 2015).

Dissolved organic carbons from *Microcystis* can be assimilated by bacteria (Casamatta and Wickstrom 2000). Cellular densities of JXJ CY 05, 11, 37, 53, and 57 in BCs, and JXJ CY 05, 11, 16, 18, 37, and 57 in TCs, and JXJ CY 53 in TCs with some other attached bacteria increased by about 1.5–100 times on day 5 of co-cultivation (Table 4), indicating that these bacteria could utilize the exudates of MF-905 for growth. However, the cellular densities of most bacteria decreased by more than 90% on day 10 of co-cultivation. Meanwhile, chl-*a* concentrations of MF-905 increased by about 0.5–1.6 times. Therefore, these bacteria could utilize cyanobacterial exudates only at the specific growth stage of MF-905. In the bio-available nitrogen-limited and tricalcium phosphate media, cellular densities of JXJ CY 53^ T^ in samples collected at the second time increased by about 80–200 times (Table 5 and 6), indicating that JXJ CY 53^ T^ could also utilize cyanobacterial exudates in the limited bio-available N and P media at specific growth stages of MF-905. Glucosidase secreted by attached bacteria participates in the utilization of soluble organic carbon from *Microcystis* (Yang et al. 2021). JXJ CY 53^ T^ has five and three gene clusters related to glucosidase and carbohydrate catabolic processes (Supplemental Table S3), respectively, which also indicated that JXJ CY 53^ T^ can potentially utilize dissolved organic carbon secreted by MF-905.

Cyanobacterial growth can be promoted by auxins secreted by bacteria (Hoke et al. 2021). Furthermore, *Microcystis* needs exogenous various vitamins such as B_1_, B7, and B_12_ for growth, of which B_12_ is required for methionine biosynthesis of the cyanobacteria (Hoke et al. 2021). Similar to other bacteria isolated from *Microcystis* (Xiao et al. 2022a,b,c), JXJ CY 53^ T^ has seven genes related to the synthesis of auxins and 11 gene clusters related to the synthesis of various vitamins (Supplemental Table S3), indicating that JXJ CY 53^ T^ can also secrete auxins to facilitate cyanobacterial growth and provide MF-905 with various vitamins.

Available N and P are the two crucial elements leading to water blooms. Secreting organic acids and phosphatase are two of the most important means of microbes dissolving insoluble phosphorus. JXJ CY 53^ T^ has 162 genes related to organic acid biosynthetic processes and four genes related to organic acid transport, indicating that it can synthesize and secrete organic acids (Supplemental Table S4). JXJ CY 53^ T^ has 26 genes related to phosphatase activity, including acid and alkaline phosphatase activity (Supplemental Table S4), indicating that its phosphatases can exhibit catalytic activity in a more extensive pH range. Presence of these genes above is consistent with the results of dissolving abilities of JXJ CY 53^ T^ on tricalcium phosphate and phytin. Therefore, JXJ CY 53^ T^ shows good dissolving activity on both insoluble organic and inorganic phosphates and can provide MF-905 with available P. This is probably the main reason why JXJ CY 53^ T^ can promote the growth of MF-905 in tricalcium phosphate medium (Table 5).

Ammonia plays an important role in the interactions of aquatic microorganisms (Cirri and Pohnert 2019). JXJ CY 53^ T^ has 18 genes involved in nitrogen fixation (Supplemental Table S4; Supplemental Fig. S4), and it grows well in nitrogen-free medium, indicating that it has a nitrogenase, which can catalyze the formation of NH_3_ from N_2_. Ammonia can dissolve in water very easily and further form ammonium (NH_4_^+^), which would be used preferentially by *Microcystis* (Yang et al. 2021). On day 14 of cultivation, the chl-*a* concentration of MF-905 co-cultured with JXJ CY 53^ T^ only decreased by 30.3% when compared with that of the previous sample collected on day 7 of cultivation, while the chl-*a* concentration of MF-905 cultured without JXJ CY 53^ T^ decreased by 56.3% when compared with that of the previous sample collected on day 7. Hence, JXJ CY 53^ T^ can offer MF-905 bio-available N and reduced the cyanobacterial death rate in the absence of bio-available N.

Associated bacteria could provide *Microcystis* with complementary carotenoid molecules (Pérez-Carrascal et al. 2021). Carotenoids play an important role in protecting chlorophyll molecules against photo-oxidative damage (Young 1991). JXJ CY 53^ T^ has four genes involved in the synthesis of carotenoids (Supplemental Table S3; Supplemental Fig. S5), including a gene encoding lycopene beta cyclase, which can catalyze the cyclization of beta rings at one or both ends of the lycopene molecule to form gamma-carotene or the bicyclic beta-carotene (Fournié and Truan 2020), respectively. Therefore, JXJ CY 53^ T^ can also potentially protect chlorophyll molecules of MF-905 against photo-oxidative damage and promote the photosynthetic efficiency of the cyanobacterium, which is probably another reason of JXJ CY 53^ T^ promoting the growth of MF-905.

### Dynamic interaction relationship of the co-culture systems

The variations of environmental conditions and growth stages would change the interactions of algae and its attached bacteria (Cooper and Smith 2015). This phenomenon was also clearly observed in the interactions between cyanobacteria and their attached bacteria before (Xiao et al. 2022a,b,c) and also in this study. For examples, JXJ CY 05 and 18 exhibited no effects on the cyanobacterial growth on day 5 of cultivation, but inhibited the cyanobacterial growth on day 10 of cultivation (*p* < 0.05, *p* < 0.01); JXJ CY 11 and 57 inhibited the cyanobacterial growth on day 5 of cultivation (*p* < 0.01), but the inhibition vanished on day 10 of cultivation; inhibitory rates of JXJ CY 37 on MF-905 increased during the test time; TC with JXJ CY 28 + 53 showed no promotion on the cyanobacterial growth on day 5 of cultivation, but promoted the cyanobacterial growth significantly on day 10 of cultivation (*p* < 0.01) (Fig. 3); cellular densities of many bacteria co-cultured with MF-905 in BG11 increased (*p* < 0.01) on day 5 of cultivation, but decreased (*p* < 0.01) on day 10 of cultivation (Table 4); cellular densities of JXJ CY 53^ T^ co-cultured with MF-905 in revised BG11 media decreased (*p* < 0.01) in the samples collected at the first time, but increased (*p* < 0.01) in the samples collected at the second time (Table 5 and 6), etc. A similar phenomenon was also found regarding the concentrations of MC-LR (Fig. 4, Table 5 and 6). Therefore, the interactions between *Microcystis* and its attached bacteria were not static, but varied with culture time.

Data in Figs. 3 and Fig. 4 and Table 4 showed that the inoculations of another attached bacterium into BC of a bacterium probably led to entirely different effects on the growths of both cyanobacterium and bacteria, and the concentrations of MC-LR, similar to the results in a previous study (Xiao et al. 2022c). Therefore, cyanobacterial growth was influenced not only by attached bacteria, but also by the interactions of cyanobacteria and other attached bacteria, and vice versa for the growth of attached bacteria. Therefore, the interactions of *Microcystis* and its attached bacteria were extremely complex.

### The possible way of healthy MF-905 controlling its attached bacteria

Similar to macroalgae (Kouzuma and Watanabe 2015), heathy *Microcystis* seems able to control its attached bacteria to avoid competition for nutrients (Xiao et al. 2022a,b,c; Zhang et al. 2016b). Extracts from *M. aeruginosa* showed inhibitory activities on some of its attached bacteria (Casamatta and Wickstrom 2000) and many other bacteria such as *Escherichia coli* (Ostensvik et al. 1998; Valdor and Aboal 2007), *Streptoverticillium* (Valdor and Aboal 2007), *Bacillus subtilis*, *Bacillus cereus*, and *Aeromonas hydrophila* (Ostensvik et al. 1998). Moreover, microcystins, including MC-LR, MC-RR, and MC-YR, were also proved to have antibacterial activity. Ostensvik et al. (1998) found that MCs showed no inhibitory activities on *E. coli*, *B. subtilis*, *B. cereus*, and *A. hydrophila* at 1–8 μg/mL, while Valdor and Aboal (2007) found that MC-YR showed inhibitory activity on *Streptoverticillium* at 12.5 μg/mL, MC-RR and MC-LR showed inhibitory effect on *Streptoverticillium* at 25 μg/mL, and MC-LR showed inhibitory activities on *E. coli* at 5 μg/mL. But, are MCs these specific chemicals of *Microcystis* controlling its attached bacteria? MC-LR accounts for more than 57% of MCs generated in *M. aeruginosa* cultured in laboratory (Liu et al. 2012) and 45.5–99.8% of MCs in the natural water blooms (Vasconcelos et al. 1996). According to our results, the concentrations of E-MC-LR, I-MC-LR, and total MC-LR were 0.014–0.32, 0.45–1.24 and 0.47–1.38 μg/mL, respectively, which indicated that the concentrations of MCs in the samples hardly meet the concentrations used in the references (Ostensvik et al. 1998; Valdor and Aboal 2007). Therefore, MCs could not be the specific chemicals of cyanobacteria controlling their attached bacteria. This was further verified by the results of antibacterial assays. Fraction III, containing MC-LR at 4 μg/disk, showed almost no inhibitory activities on those strains, while fraction I and II, especially II, showed stronger and broader-spectrum antibacterial activity. Therefore, fraction II contains one of the main specific chemicals of MF-905 controlling its attached bacteria.

Data in Table 4 indicated that the antibacterial component in MF-905 cultures increased with the culture times, and finally exhibited inhibitory activity. Metabolites from the cyanobacterium exhibited almost no inhibitory activity on JXJ CY 28 on plate. However, JXJ CY 28 co-cultured with MF-905 was inhibited on both days 5 and 10 of cultivation. Similarly, extract of the cyanobacterium also exhibited almost no inhibitory activity on JXJ CY 05, 11, 18, and 57. However, the cellular densities of those bacteria decreased significantly in the samples collected at the second time (Table 4), indicating that different antibacterial components were probably synthesized and secreted in these samples. However, in tricalcium phosphate medium, the cellular density of JXJ CY 53^ T^ increased more than 80 times on day 18 of cultivation compared with that of the previous sample (Table 5), indicating that the concentrations of the antibacterial component in MF-905 culture did not increase, even probably decreased, though the chl-*a* concentration increased by 30% in the meantime. Only enough biomass of JXJ CY 53^ T^ can provide the cyanobacterium with enough available P in tricalcium phosphate medium. This was probably the main reason that the cellular density of JXJ CY 53^ T^ increased on day 18 of cultivation. Therefore, MF-905 seemed able to adjust the synthesis and secretion of the specific antibacterial component according to the nutritional demands.

The illumination of the compounds of healthy MF-905 controlling its attached bacteria would play an important role in throwing light on the interactions between *Microcystis* and its attached bacteria, which would probably further provide clues of controlling cyanobacterial blooms. Therefore, more studies should be focused on these issues above.

## Supplementary Information

Below is the link to the electronic supplementary material.

Supplementary file1 (PDF 865 KB)

## Data Availability

Some data presented in this study are contained in the published paper and its supplementary materials, and other data can be found in online repositories.
