# Supplementary material for: Sphingomonas lacusdianchii sp. nov., an attached bacterium inhibited by metabolites from its symbiotic cyanobacterium
Source: Appl Microbiol Biotechnol. 2024 Apr 25;108:309. doi: 10.1007/s00253-024-13081-x (PMC11045571; doi:10.1007/s00253-024-13081-x)
Supplement: Supplementary file 1 [file 253_2024_13081_MOESM1_ESM.pdf]

Sphingomonas lacusdianchii sp. nov., an attached bacterium inhibited by metabolites from its symbiotic cyanobacterium

Xin Wang [1], Yao Xiao [1], Yang Deng [2], Xue Sang[1], Qing-Lin Deng[1], Le Wang[1], Yi-Wen Yang [1], Bing-Huo Zhang [1*], Yu-Qin Zhang [2*]

[1] College of Pharmacy and Life Science, Jiujiang University, Jiujiang, 332000, China

[2] Institute of Medicinal Biotechnology, Chinese Academy of Medical Sciences & Peking Union Medical College, Beijing 100050, China

Author for correspondence:

Bing-Huo Zhang

Tel & Fax: +86-792-8565939

E-Mail: binghuozh@126.com (B. Zhang)

Yu-Qin Zhang

Tel & Fax: +86-10-83167110

E-Mail: zhyuqin@126.com (Y. Zhang)

Xin Wang, Yao Xiao, Yang Deng contributed equally to this work.

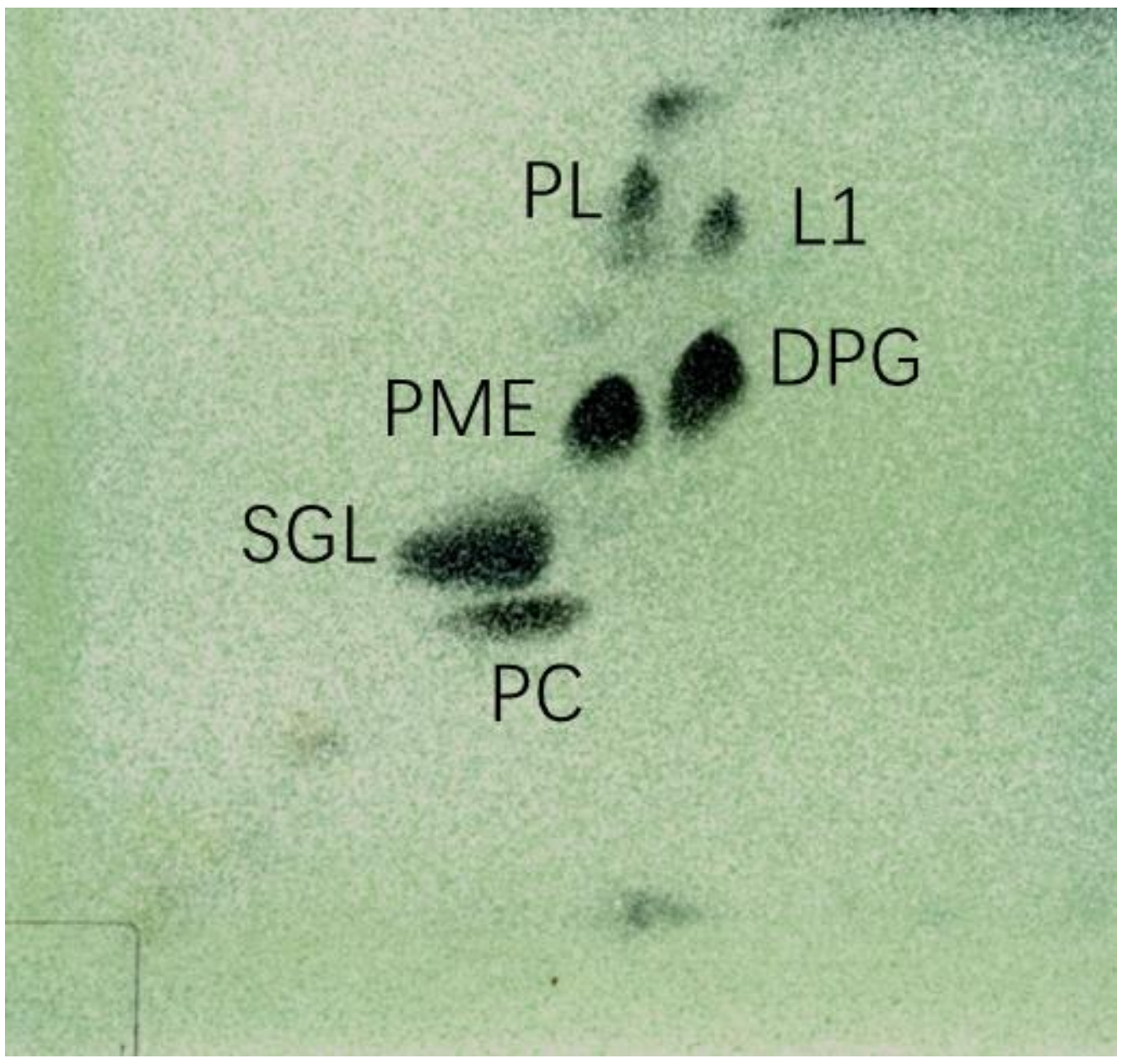

Fig. S1 The polar lipids of strain JXJ CY 53^T as revealed by two-dimensional TLC.

DPG, diphosphatidylglycerol; PME, phosphatidylmethylethanolamine; SGL, sphingoglycolipid; PC, phosphatidylcholine; PL, an unidentified phospholipid; L1, unidentified lipid.

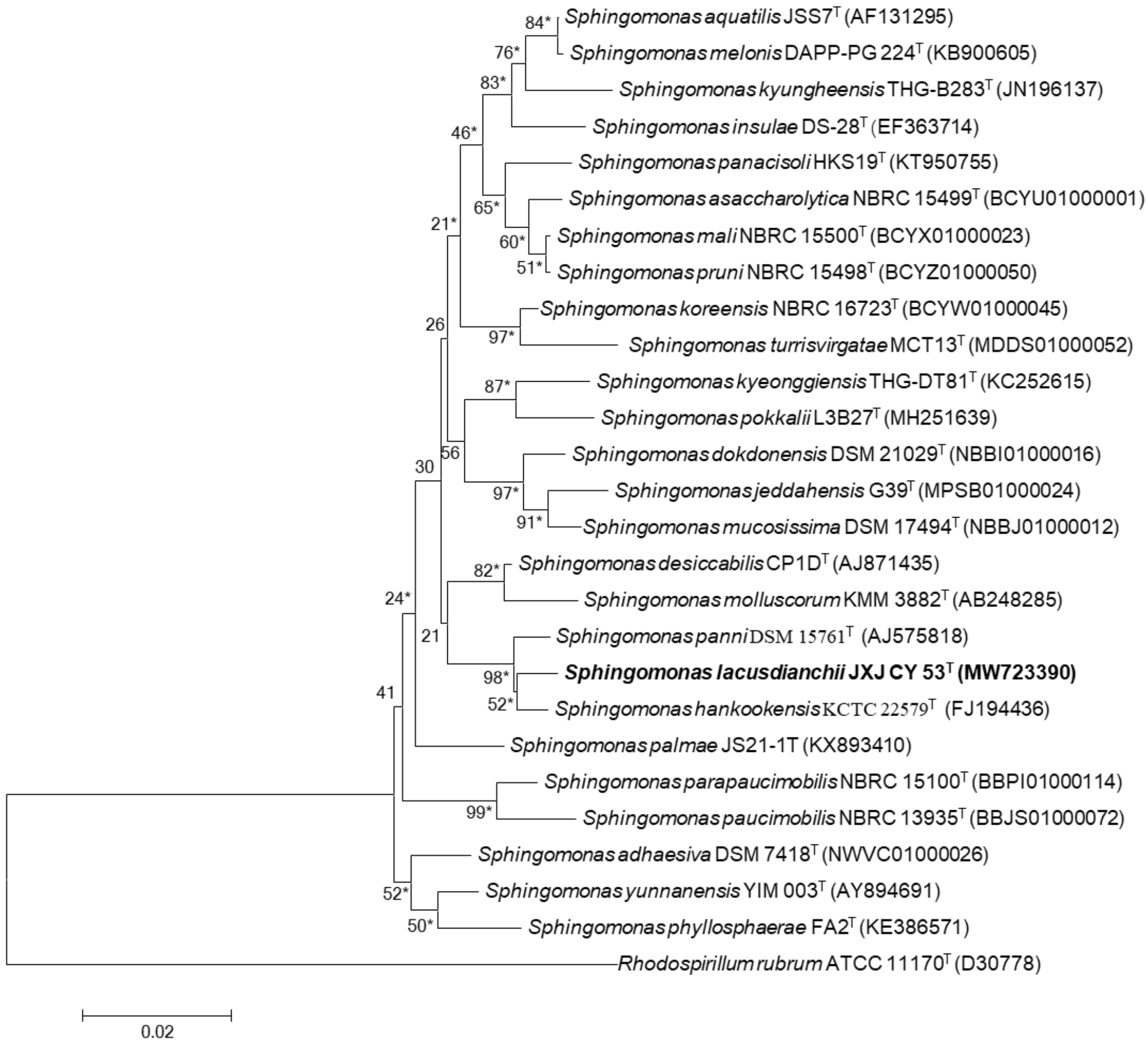

Fig. S2 Maximum-likelihood phylogenetic tree based on 16S rRNA gene sequences of strain JXJ CY 53^T and its closest relative species of the genus *Sphingomonas*. * indicated clades that were conserved in neighbour-joining, maximum-likelihood and maximum-parsimony trees. Bar, 0.02 changes per nucleotide position.

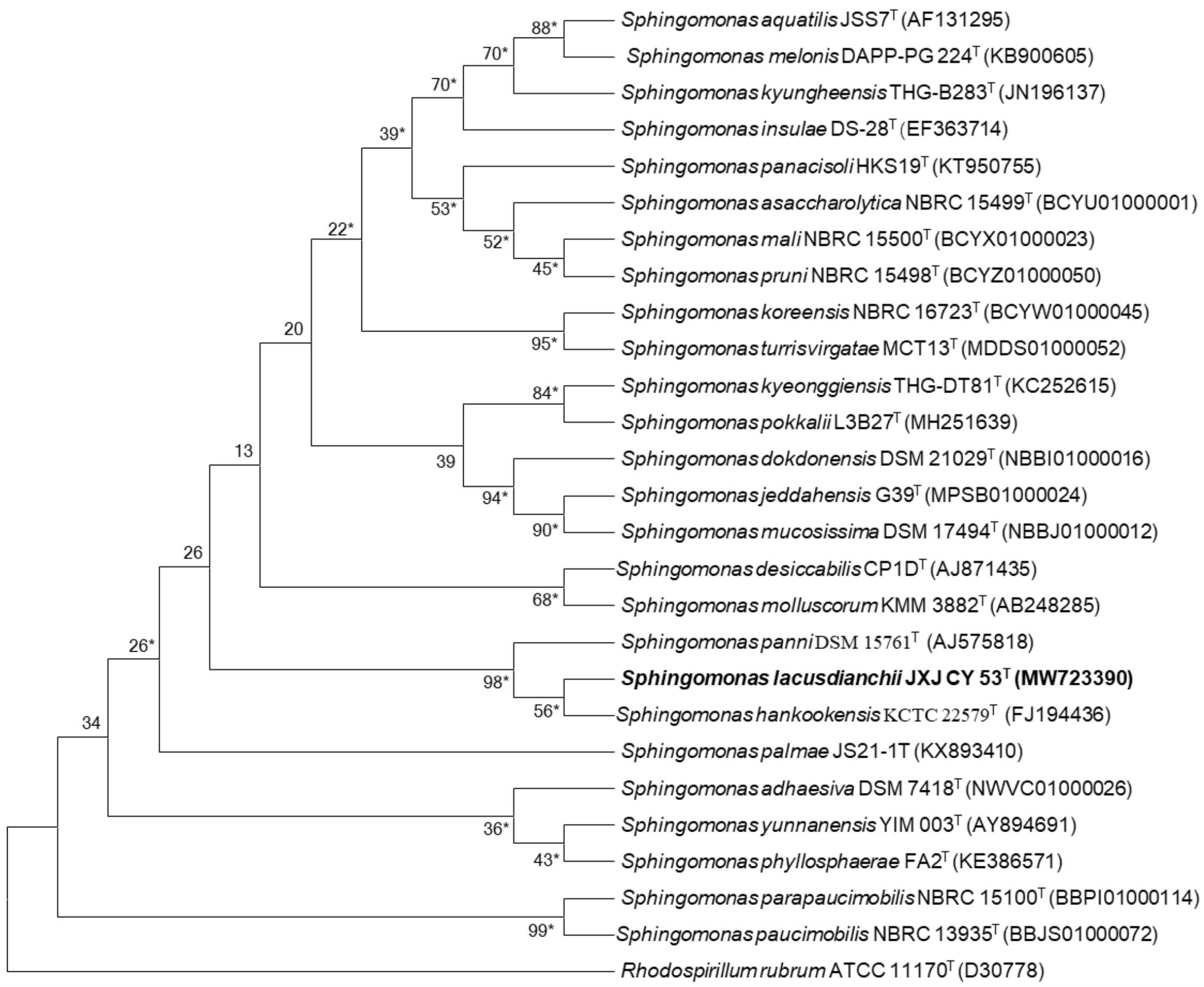

Fig. S3 Maximum-parsimony phylogenetic tree based on 16S rRNA gene sequences of strain JXJ CY 53^T and its closest relative species of the genus *Sphingomonas*. * indicated clades that were conserved in neighbour-joining, maximum-likelihood and maximum-parsimony trees.

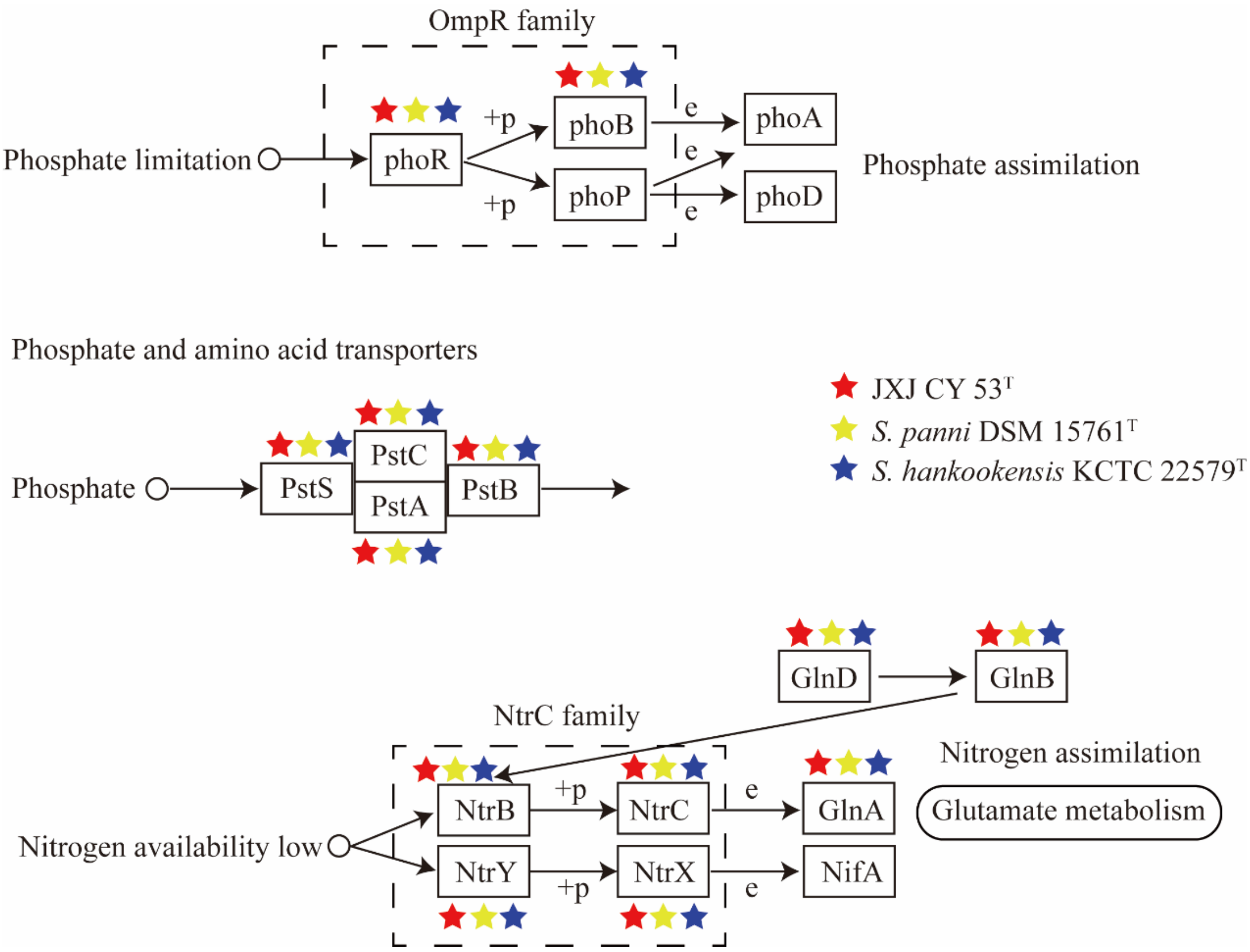

Fig. S4 The KEGG pathway for phosphate assimilation and nitrogen fixation of the strains JXJ CY 53^{T}, *S. panni* DSM 15761^{T} and *S. hankookensis* KCTC 22579^{T}.

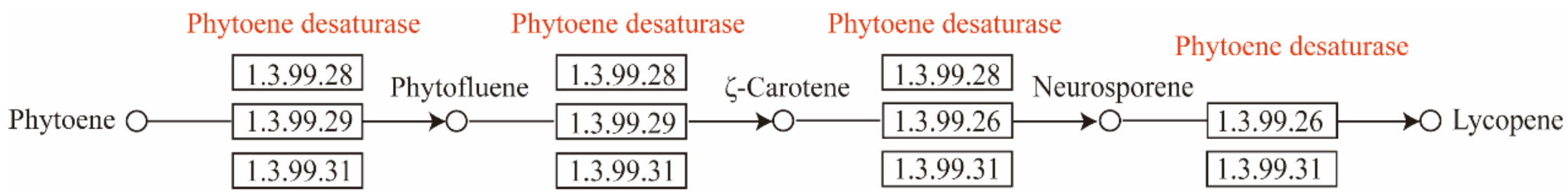

Fig. S5 Putative overview of four-step desaturations which desaturated phytoene to lycopene of the strains JXJ CY 53^T, *S. panni* DSM 15761^T and *S. hankookensis* KCTC 22579^T.

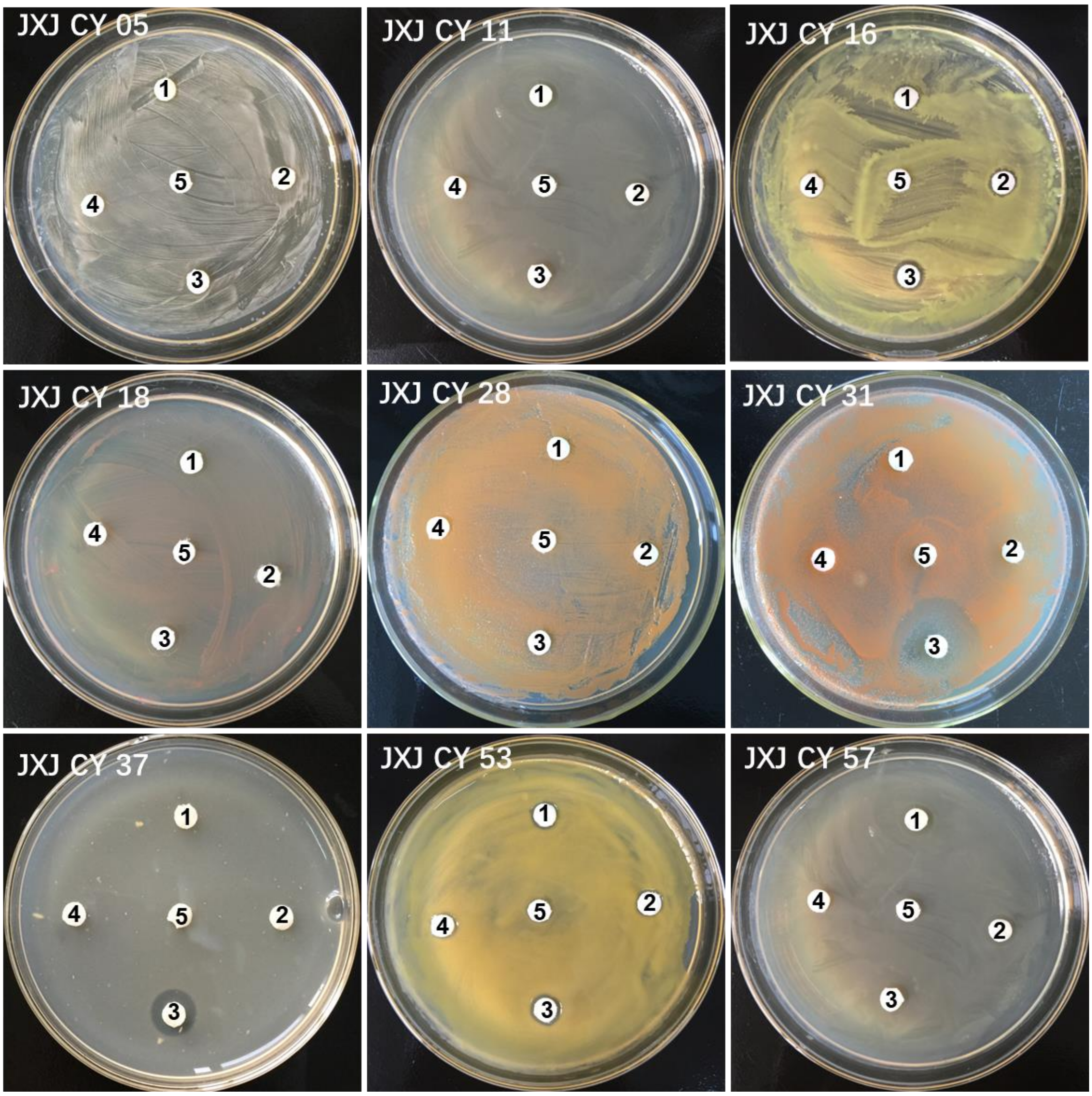

Fig. S6 Inhibitory activity of extracts from MF-905 on nine attached bacterial strains. 1, 2, 3, 4, and 5 indicated the total extract from Mf-905, in addition to fractions I, II, III, and IV, respectively. Only fraction III contained MC-LR.

Table S1 Cellular fatty acid profile of strain JXJ CY 53^T and related reference strains

Fatty acid	JXJ CY 53^T	*S. panni* DSM 15761^T (Busse et al. 2005)	*S. hankookensis* KCTC 22579^T (Yoon et al. 2009)
$C_{14:0}$	0.99	1.1	1.1
$C_{15:0}$	-	1.1	0.5
$C_{16:0}$	14.56	17.8	11.5
$C_{17:0}$	0.59	ND	ND
$C_{18:0}$	0.32	ND	ND
$C_{16:1}\omega7c/_{16:1}\omega6c$	10.78	ND	ND
$C_{16:1}\omega7c$/ iso $C_{15:0}$ 2-OH	-	17.1	16.2
$C_{16:1}\omega5c$	4.74	2.1	5.4
$C_{17:1}\omega8c$	0.94	ND	0.5
$C_{17:1}\omega6c$	7.60	3.6	3.7
$C_{18:1}\omega7c$	48.74	51.3	53.9
$C_{18:1}\omega5c$	1.75	0.7	1.2
$C_{14:0}$ 2-OH	2.58	5.3	5.6
$C_{15:0}$ 2-OH	0.41	ND	0.2
11-Methyl $C_{18:1}\omega7c$	4.62	ND	ND

-, No related fatty acids detected; ND, No data.

Table S2 The possibly partial important genes or gene clusters for strain JXJ CY 53^T to adapt to the ecology of MF-905

Level	GO	Term	Ontology	Gene_num	Gene_list
3	GO:0052192	Movement in environment of other organism involved in symbiotic interaction	Biological_process	10	PROKKA_01138, PROKKA_00237, PROKKA_00152, PROKKA_04210, PROKKA_00903, PROKKA_02877, PROKKA_02157, PROKKA_04445, PROKKA_00007, PROKKA_02486
4	GO:0051828	Entry into other organism involved in symbiotic interaction	Biological_process	9	PROKKA_01138, PROKKA_00237, PROKKA_00152, PROKKA_04210, PROKKA_00903, PROKKA_02877, PROKKA_02157, PROKKA_04445, PROKKA_00007
4	GO:0052173	Response to defenses of other organism involved in symbiotic interaction	Biological_process	4	PROKKA_02934, PROKKA_02032, PROKKA_01408, PROKKA_02419
4	GO:0052195	Movement on or near other organism involved in symbiotic interaction	Biological_process	1	PROKKA_02486
4	GO:0052216	Chemotaxis in environment of other organism involved in symbiotic interaction	Biological_process	1	PROKKA_02486
5	GO:0051806	Entry into cell of other organism involved in symbiotic interaction	Biological_process	9	PROKKA_01138, PROKKA_00237, PROKKA_00152, PROKKA_04210, PROKKA_00903, PROKKA_02877, PROKKA_02157, PROKKA_04445, PROKKA_00007
5	GO:0051807	Evasion or tolerance of defense response of other organism involved in symbiotic interaction	Biological_process	1	PROKKA_02934

5	GO:0051832	Avoidance of defenses of other organism involved in symbiotic interaction	Biological_process	1	PROKKA_02934
5	GO:0052243	Chemotaxis on or near other organism involved in symbiotic interaction	Biological_process	1	PROKKA_02486
5	GO:0052564	Response to immune response of other organism involved in symbiotic interaction	Biological_process	4	PROKKA_02934, PROKKA_02032, PROKKA_01408, PROKKA_02419
6	GO:0051805	Evasion or tolerance of immune response of other organism involved in symbiotic interaction	Biological_process	1	PROKKA_02934
6	GO:0051834	Evasion or tolerance of defenses of other organism involved in symbiotic interaction	Biological_process	1	PROKKA_02934
6	GO:0052143	Chemotaxis on or near host involved in symbiotic interaction	Biological_process	1	PROKKA_02486
6	GO:0052550	Response to defense-related reactive oxygen species production by other organism involved in symbiotic interaction	Biological_process	1	PROKKA_02934

6	GO:0052551	Response to defense-related nitric oxide production by other organism involved in symbiotic interaction	Biological_process	1	PROKKA_02934
6	GO:0052572	Response to host immune response	Biological_process	4	PROKKA_02934, PROKKA_02032, PROKKA_01408, PROKKA_02419
7	GO:0020012	Evasion or tolerance of host immune response	Biological_process	1	PROKKA_02934
7	GO:0052376	Evasion or tolerance by organism of nitric oxide produced by other organism involved in symbiotic interaction	Biological_process	1	PROKKA_02934
7	GO:0052385	Evasion or tolerance by organism of reactive oxygen species produced by other organism involved in symbiotic interaction	Biological_process	1	PROKKA_02934

Table S3 The possibly partial important genes and gene clusters related to the signal transductions and nutrient exchanges between strain JXJ CY 53^{T} and MF-905

Level	GO	Term	Ontology	Gene_num	Gene_list
3	GO:0043190	ATP-binding cassette (ABC) transporter complex	Cellular_component	4	PROKKA_02408, PROKKA_01215, PROKKA_03018, PROKKA_00590
3	GO:0015627	Type II protein secretion system complex	Cellular_component	8	PROKKA_00602, PROKKA_00584, PROKKA_00577, PROKKA_00583, PROKKA_00576, PROKKA_00580, PROKKA_00581, PROKKA_00582
3	GO:0030256	Type I protein secretion system complex	Cellular_component	1	PROKKA_03647
3	GO:0030257	Type III protein secretion system complex	Cellular_component	1	PROKKA_00980
5	GO:0009306	Protein secretion	Biological_process	23	PROKKA_03648, PROKKA_00583, PROKKA_00459, PROKKA_00576, PROKKA_01857, PROKKA_00580, PROKKA_00980, PROKKA_03594, PROKKA_03294, PROKKA_00602, PROKKA_00584, PROKKA_00577, PROKKA_03855, PROKKA_04123, PROKKA_00458, PROKKA_00581, PROKKA_00601, PROKKA_01436, PROKKA_00987, PROKKA_00988, PROKKA_00990, PROKKA_03647, PROKKA_00582

6	GO:0015628	Protein secretion by the type II secretion system	Biological_process	8	PROKKA_00602, PROKKA_00584, PROKKA_00577, PROKKA_00583, PROKKA_00576, PROKKA_00580, PROKKA_00581, PROKKA_00582
6	GO:0030253	Protein secretion by the type I secretion system	Biological_process	1	PROKKA_03647
6	GO:0030254	Protein secretion by the type III secretion system	Biological_process	1	PROKKA_00980
6	GO:0009734	Auxin mediated signaling pathway	Biological_process	1	PROKKA_01128
6	GO:0009785	Blue light signaling pathway	Biological_process	5	PROKKA_00834, PROKKA_00664, PROKKA_01061, PROKKA_03349, PROKKA_03413
6	GO:0015926	Glucosidase activity	Molecular_function	13	PROKKA_00385, PROKKA_01314, PROKKA_04116, PROKKA_01589, PROKKA_00283, PROKKA_00208, PROKKA_01606, PROKKA_01981, PROKKA_03467, PROKKA_00271, PROKKA_02311, PROKKA_00676, PROKKA_01879

7	GO:0004558	Alpha-glucosidase activity	Molecular_function	6	PROKKA_02311, PROKKA_00385, PROKKA_01314, PROKKA_01589, PROKKA_01981, PROKKA_00271
7	GO:0004575	Sucrose alpha-glucosidase activity	Molecular_function	2	PROKKA_02311, PROKKA_01314
7	GO:0008422	Beta-glucosidase activity	Molecular_function	7	PROKKA_00676, PROKKA_04116, PROKKA_00283, PROKKA_01879, PROKKA_00208, PROKKA_01606, PROKKA_03467
8	GO:0032450	Maltose alpha-glucosidase activity	Molecular_function	4	PROKKA_01589, PROKKA_00385, PROKKA_01981, PROKKA_00271
5	GO:0016052	Carbohydrate catabolic process	Biological_process	85	PROKKA_01583, PROKKA_01882, PROKKA_00888, PROKKA_02864, PROKKA_02647, PROKKA_03289, PROKKA_00249, PROKKA_01194, PROKKA_00367, PROKKA_04087, PROKKA_04151, PROKKA_03150, PROKKA_00319, PROKKA_00260, PROKKA_03628, PROKKA_00448, PROKKA_00676, PROKKA_04012, PROKKA_00311, PROKKA_00241, PROKKA_01535, PROKKA_03287, PROKKA_02599, PROKKA_00552, PROKKA_01737, PROKKA_01996, PROKKA_01606, PROKKA_01659, PROKKA_03503, PROKKA_04086, PROKKA_03381, PROKKA_04001, PROKKA_00268, PROKKA_01002, PROKKA_03133, PROKKA_01161, PROKKA_04256, PROKKA_02751, PROKKA_03203, PROKKA_02859, PROKKA_03610, PROKKA_03131, PROKKA_03801, PROKKA_01067, PROKKA_02579, PROKKA_02609, PROKKA_02436, PROKKA_02537, PROKKA_00629, PROKKA_03467, PROKKA_03663, PROKKA_02531, PROKKA_00617, PROKKA_00369, PROKKA_03117, PROKKA_00828, PROKKA_04257, PROKKA_00656, PROKKA_03672, PROKKA_01988,

5	GO:0044275	Cellular carbohydrate catabolic process	Biological_process	28	PROKKA_00657, PROKKA_03263, PROKKA_01580, PROKKA_00923, PROKKA_03290, PROKKA_01314, PROKKA_00312, PROKKA_01584, PROKKA_01973, PROKKA_03796, PROKKA_00364, PROKKA_01150, PROKKA_01761, PROKKA_01558, PROKKA_02583, PROKKA_03379, PROKKA_00280, PROKKA_00370, PROKKA_00086, PROKKA_02591, PROKKA_03051, PROKKA_01070, PROKKA_00896, PROKKA_04007, PROKKA_04113
6	GO:0044724	Single-organism carbohydrate catabolic process	Biological_process	61	PROKKA_03801, PROKKA_01583, PROKKA_01314, PROKKA_02537, PROKKA_00552, PROKKA_01606, PROKKA_01194, PROKKA_03467, PROKKA_01973, PROKKA_04087, PROKKA_03663, PROKKA_02531, PROKKA_01150, PROKKA_03150, PROKKA_00617, PROKKA_04086, PROKKA_00676, PROKKA_01558, PROKKA_00268, PROKKA_03117, PROKKA_00828, PROKKA_00656, PROKKA_04012, PROKKA_03672, PROKKA_01161, PROKKA_00657, PROKKA_03263, PROKKA_00896 PROKKA_01583, PROKKA_02864, PROKKA_03289, PROKKA_01194, PROKKA_00367, PROKKA_04087, PROKKA_04151, PROKKA_03150, PROKKA_00319, PROKKA_03628, PROKKA_00676, PROKKA_04012, PROKKA_00311, PROKKA_03287, PROKKA_02599, PROKKA_00552, PROKKA_01737, PROKKA_01606, PROKKA_01659, PROKKA_04086, PROKKA_00268, PROKKA_01002, PROKKA_01161, PROKKA_04256, PROKKA_02751, PROKKA_03203, PROKKA_01067, PROKKA_03801, PROKKA_02579, PROKKA_02609, PROKKA_02436, PROKKA_02537, PROKKA_00629, PROKKA_03467, PROKKA_02531, PROKKA_03663, PROKKA_00617, PROKKA_00369, PROKKA_03117, PROKKA_04257, PROKKA_00828, PROKKA_00656, PROKKA_03672, PROKKA_00657, PROKKA_03263, PROKKA_00923, PROKKA_03290, PROKKA_01314, PROKKA_00312, PROKKA_01584, PROKKA_03796, PROKKA_01973, PROKKA_00364, PROKKA_01150, PROKKA_01761, PROKKA_01558, PROKKA_02583, PROKKA_00280, PROKKA_00370, PROKKA_01070, PROKKA_00896

6	GO:0080030	Methyl indole-3-acetate esterase activity	Molecular_function	2	PROKKA_03557, PROKKA_03772
8	GO:0006596	Polyamine biosynthetic process	Biological_process	5	PROKKA_00082, PROKKA_00034, PROKKA_03587, PROKKA_04150, PROKKA_04148
7	GO:0000162	Tryptophan biosynthetic process	Biological_process	4	PROKKA_01947, PROKKA_03386, PROKKA_03385, PROKKA_02438
4	GO:0045436	Lycopene beta cyclase activity	Molecular_function	1	PROKKA_03866
5	GO:0016117	Carotenoid biosynthetic process	Biological_process	4	PROKKA_03867, PROKKA_03866, PROKKA_04377, PROKKA_03869
3	GO:0051183	Vitamin transporter activity	Molecular_function	11	PROKKA_02637, PROKKA_03155, PROKKA_00639, PROKKA_00543, PROKKA_01010, PROKKA_01227, PROKKA_01603, PROKKA_03103, PROKKA_03014, PROKKA_01612, PROKKA_03844
3	GO:0009349	Riboflavin synthase complex	Cellular_component	1	PROKKA_04389
4	GO:0004076	Biotin synthase activity	Molecular_function	1	PROKKA_00387

4	GO:0051180	Vitamin transport	Biological_process	1	PROKKA_00635
4	GO:0015235	Cobalamin transporter activity	Molecular_function	11	PROKKA_02637, PROKKA_03155, PROKKA_00639, PROKKA_00543, PROKKA_01010, PROKKA_01227, PROKKA_01603, PROKKA_03103, PROKKA_03014, PROKKA_01612, PROKKA_03844
5	GO:0004746	Riboflavin synthase activity	Molecular_function	1	PROKKA_04387
5	GO:0009110	Vitamin biosynthetic process	Biological_process	43	PROKKA_00379, PROKKA_00631, PROKKA_00995, PROKKA_00638, PROKKA_02743, PROKKA_03124, PROKKA_03387, PROKKA_02524, PROKKA_04338, PROKKA_00380, PROKKA_03917, PROKKA_04237, PROKKA_03752, PROKKA_00387, PROKKA_04386, PROKKA_00632, PROKKA_00633, PROKKA_01101, PROKKA_01741, PROKKA_03722, PROKKA_00277, PROKKA_02746, PROKKA_00363, PROKKA_02467, PROKKA_02720, PROKKA_02406, PROKKA_00630, PROKKA_03872, PROKKA_00111, PROKKA_00637, PROKKA_04388, PROKKA_03934, PROKKA_04387, PROKKA_00381, PROKKA_02967, PROKKA_00293, PROKKA_04325, PROKKA_04360, PROKKA_02024, PROKKA_00641, PROKKA_03738, PROKKA_04079, PROKKA_04389
5	GO:0015889	Cobalamin transport	Biological_process	1	PROKKA_00635
6	GO:0042364	Water-soluble vitamin biosynthetic process	Biological_process	43	PROKKA_00379, PROKKA_00631, PROKKA_00995, PROKKA_00638, PROKKA_02743, PROKKA_03124, PROKKA_03387, PROKKA_02524, PROKKA_04338, PROKKA_00380, PROKKA_03917, PROKKA_04237, PROKKA_03752, PROKKA_00387, PROKKA_04386, PROKKA_00632, PROKKA_00633, PROKKA_01101, PROKKA_01741, PROKKA_03722, PROKKA_00277, PROKKA_02746, PROKKA_00363, PROKKA_02467, PROKKA_02720,

					PROKKA_02406, PROKKA_00630, PROKKA_03872, PROKKA_00111, PROKKA_00637, PROKKA_04388, PROKKA_03934, PROKKA_04387, PROKKA_00381, PROKKA_02967, PROKKA_00293, PROKKA_04325, PROKKA_04360, PROKKA_02024, PROKKA_00641, PROKKA_03738, PROKKA_04079, PROKKA_04389
6	GO:0009102	Biotin biosynthetic process	Biological_process	5	PROKKA_00379, PROKKA_02406, PROKKA_00381, PROKKA_00387, PROKKA_00380
7	GO:0009236	Cobalamin biosynthetic process	Biological_process	13	PROKKA_00631, PROKKA_00995, PROKKA_00638, PROKKA_00630, PROKKA_03124, PROKKA_02524, PROKKA_00637, PROKKA_02967, PROKKA_00632, PROKKA_00633, PROKKA_00641, PROKKA_03722, PROKKA_03738
7	GO:0042819	Vitamin B6 biosynthetic process	Biological_process	4	PROKKA_00111, PROKKA_04360, PROKKA_03934, PROKKA_04237
7	GO:0009234	Menaquinone biosynthetic process	Biological_process	4	PROKKA_02521, PROKKA_01110, PROKKA_03686, PROKKA_02963
7	GO:0009231	Riboflavin biosynthetic process	Biological_process	6	PROKKA_04386, PROKKA_04325, PROKKA_04388, PROKKA_03917, PROKKA_04389, PROKKA_04387
7	GO:0009228	Thiamine biosynthetic process	Biological_process	6	PROKKA_03752, PROKKA_03872, PROKKA_04338, PROKKA_00363, PROKKA_02746, PROKKA_02467

Table S4 The possibly partial important genes or gene clusters for strain JXJ CY 53^T to provide MF-905 with available P and N

Level	GO	Term	Ontology	Gene_num	Gene_list
5	GO:0016053	Organic acid biosynthetic process	Biological_process	162	PROKKA_03876, PROKKA_00379, PROKKA_00061, PROKKA_03412, PROKKA_03291, PROKKA_00380, PROKKA_03843, PROKKA_00653, PROKKA_03152, PROKKA_03309, PROKKA_02429, PROKKA_03417, PROKKA_00863, PROKKA_00606, PROKKA_00277, PROKKA_03627, PROKKA_01943, PROKKA_03549, PROKKA_00328, PROKKA_03968, PROKKA_04238, PROKKA_02406, PROKKA_00401, PROKKA_02663, PROKKA_03933, PROKKA_01773, PROKKA_01930, PROKKA_03865, PROKKA_02639, PROKKA_03900, PROKKA_02923, PROKKA_00399, PROKKA_03932, PROKKA_00865, PROKKA_03027, PROKKA_00935, PROKKA_02009, PROKKA_00254, PROKKA_01134, PROKKA_01719, PROKKA_03283, PROKKA_03617, PROKKA_04244, PROKKA_03419, PROKKA_01836, PROKKA_02627, PROKKA_02511, PROKKA_03771, PROKKA_02997, PROKKA_03392, PROKKA_03884, PROKKA_00387, PROKKA_00022, PROKKA_01610, PROKKA_01101, PROKKA_03415, PROKKA_02987, PROKKA_02401, PROKKA_02720, PROKKA_03477, PROKKA_00420, PROKKA_02736, PROKKA_04225, PROKKA_01965, PROKKA_02428, PROKKA_02458, PROKKA_00994, PROKKA_03281, PROKKA_04403, PROKKA_03407, PROKKA_02995, PROKKA_04249, PROKKA_00913, PROKKA_01202, PROKKA_04414, PROKKA_02937, PROKKA_02968, PROKKA_02614, PROKKA_00469, PROKKA_04371, PROKKA_03385, PROKKA_00143, PROKKA_01774, PROKKA_02743, PROKKA_04050, PROKKA_02856, PROKKA_00032, PROKKA_02438, PROKKA_03643, PROKKA_02403, PROKKA_04402, PROKKA_03279, PROKKA_03284, PROKKA_03416, PROKKA_01741, PROKKA_04219, PROKKA_01551, PROKKA_02008, PROKKA_03219, PROKKA_03890, PROKKA_00453, PROKKA_03441, PROKKA_00373, PROKKA_01906, PROKKA_03396, PROKKA_00015, PROKKA_03305, PROKKA_01177, PROKKA_03934, PROKKA_02969, PROKKA_00293, PROKKA_02985, PROKKA_04245, PROKKA_03846, PROKKA_02990,

					PROKKA_02986, PROKKA_03112, PROKKA_02690, PROKKA_02974, PROKKA_00486, PROKKA_01757, PROKKA_01833, PROKKA_00866, PROKKA_04310, PROKKA_01104, PROKKA_00867, PROKKA_03387, PROKKA_02047, PROKKA_03110, PROKKA_00033, PROKKA_03988, PROKKA_00951, PROKKA_03585, PROKKA_03465, PROKKA_03750, PROKKA_04298, PROKKA_01105, PROKKA_02618, PROKKA_02409, PROKKA_03673, PROKKA_03457, PROKKA_03940, PROKKA_00327, PROKKA_04037, PROKKA_03006, PROKKA_01864, PROKKA_01131, PROKKA_03391, PROKKA_03797, PROKKA_00381, PROKKA_03029, PROKKA_01947, PROKKA_00489, PROKKA_00918, PROKKA_02024, PROKKA_04327, PROKKA_04079, PROKKA_04377, PROKKA_02455, PROKKA_03751, PROKKA_03386, PROKKA_04287
4	GO:0015849	Organic acid transport	Biological_process	4	PROKKA_03036, PROKKA_03478, PROKKA_04070, PROKKA_00493
5	GO:0005342	Organic acid transmembrane transporter activity	Molecular_function	15	PROKKA_03500, PROKKA_01546, PROKKA_03380, PROKKA_03619, PROKKA_02749, PROKKA_00617, PROKKA_03138, PROKKA_03769, PROKKA_00570, PROKKA_01987, PROKKA_01588, PROKKA_04018, PROKKA_03483, PROKKA_01013, PROKKA_03098
6	GO:0016791	Phosphatase activity	Molecular_function	26	PROKKA_03874, PROKKA_01756, PROKKA_01585, PROKKA_01578, PROKKA_02697, PROKKA_03900, PROKKA_03706, PROKKA_03149, PROKKA_03072, PROKKA_04370, PROKKA_00624, PROKKA_04075, PROKKA_00359, PROKKA_02004, PROKKA_03896, PROKKA_02860, PROKKA_01080, PROKKA_04253, PROKKA_01654, PROKKA_00918, PROKKA_04245, PROKKA_02655, PROKKA_02992, PROKKA_03419, PROKKA_03032, PROKKA_02313
7	GO:0003993	Acid phosphatase activity	Molecular_function	5	PROKKA_04253, PROKKA_01756, PROKKA_03072, PROKKA_02992, PROKKA_02004

7	GO:0004035	Alkaline phosphatase activity	Molecular_function	1	PROKKA_01654
5	GO:0009877	Nodulation	Biological_process	10	PROKKA_03281, PROKKA_01154, PROKKA_03661, PROKKA_01806, PROKKA_01056, PROKKA_00028, PROKKA_04290, PROKKA_01165, PROKKA_02505, PROKKA_02453
5	GO:0009399	nitrogen fixation	Biological_process	18	PROKKA_04330, PROKKA_01127, PROKKA_00663, PROKKA_00137, PROKKA_02592, PROKKA_00662, PROKKA_03707, PROKKA_03361, PROKKA_03824, PROKKA_00136, PROKKA_00138, PROKKA_02234, PROKKA_01758, PROKKA_02233, PROKKA_00660, PROKKA_02235, PROKKA_00135, PROKKA_01757
